# Desacetyl-α-MSH and α-MSH have sex specific interactions with diet to influence mouse gut morphology, metabolites and microbiota

**DOI:** 10.1038/s41598-020-75786-z

**Published:** 2020-11-03

**Authors:** Bo Sun, Tommi Vatanen, Thilini N. Jayasinghe, Elizabeth McKenzie, Rinki Murphy, Justin M. O’Sullivan

**Affiliations:** 1 Department of Physiology, Faculty of Medical and Health Sciences, University of Auckland, Auckland, New Zealand; 2 The Liggins Institute, University of Auckland, 85 Park Road, Grafton, Auckland, 1142 New Zealand; 3 School of Medicine, Faculty of Medical and Health Sciences, University of Auckland, Auckland, New Zealand; 4 Department of Medicine, Maurice Wilkins Centre for Molecular Biodiscovery, The University of Auckland, 85 Park Road, Grafton, Auckland, 1142 New Zealand

**Keywords:** Microbiome, Obesity

## Abstract

The melanocortin peptides have an important role in regulating body weight and appetite. Mice that lack the desacetyl-α-MSH and α-MSH peptides (*Pomc*^tm1/tm1^) develop obesity. This effect is exacerbated by a high fat diet (HFD). However, development of obesity in female *Pomc*^tm1/tm1^ mice during chronic HFD conditions is not fully accounted for by the increased energy intake. We hypothesized that the protection against chronic HFD-induced obesity imparted by MSH peptides in females is mediated by sex-specific alterations in the gut structure and gut microbiota. We determined that female WT mice had reduced jejunum villus length and increased crypt depth in response to chronic HFD. WT males and *Pomc*^tm1/tm1^ mice lacked this adaptation to a chronic HFD. Both *Pomc*^tm1/tm1^ genotype and chronic HFD were significantly associated with gut microbiota composition. Sex-specific associations between *Pomc*^tm1/tm1^ genotype and gut microbiota were observed in the presence of a chronic HFD. *Pomc*^tm1/tm1^ females had significantly reduced fecal acetate and propionate concentrations when compared to WT females. We conclude that MSH peptides influence jejunum villus length, crypt depth and the structure of the gut microbiota. These effects favor reduced nutrient absorption and occur in addition to the recognized roles of desacetyl-α-MSH and α-MSH peptides in appetite control.

## Introduction

The melanocortin system is central to body weight regulation, influencing feeding behavior, energy expenditure, glucose control, and lipid metabolism^1,2^. In a high-energy diet environment, the melanocortin system is understood to limit feeding behavior and to increase energy expenditure to maintain energy homeostasis and counteract obesity^3^. Identifying the pathways by which this occurs could lead to novel treatments for human obesity.

Pro-opiomelanocortin (POMC) is a precursor peptide in the melanocortin system. POMC is processed into α-, β-, ϒ-melanocyte-stimulating hormone (α-, β-, ϒ-MSH, respectively), adrenocorticotropic hormone, and β-endorphin^4^. Genetic defects in the POMC gene leads to early-onset obesity with hyperphagia, which is thought to be a result of inadequate production of both α- and β-MSH. However, mice with a specific mutation in the *Pomc* gene (C57BL/6J; *Pomc*^tm1/tm1^ mice) who are unable to synthesize desacetyl-α-MSH and α-MSH have been shown to develop obesity on a chow diet. This obese phenotype was exacerbated with acute and chronic exposure to HFD^5,6^.

There is clear sexual dimorphism in body fat distribution among men and women. The propensity to obesity also appears to be greater among women^7^, with international rates of obesity being higher in women than in men. Mice exhibit sexually dimorphic changes in response to HF diets^6^, and provide a good model to study this phenomenon. Specifically, C57BL/6J males become obese on a chronic HF diet. By contrast, C57BL/6J female mice are protected from diet-induced obesity, partly due to a reduction in food intake^6^. Notably, this sexually dimorphic response to the HF diet is not observed in *Pomc*^tm1/tm1^ mice^6^. Instead, male and female *Pomc*^tm1/tm1^ mice develop obesity on a normal chow diet^5^, and the level of obesity is exacerbated by a chronic HF diet^6^. Male *Pomc*^tm1/tm1^ mice significantly increased energy intake, which resulted in obesity^6^. Notably, the obesity phenotype observed in female *Pomc*^tm1/tm1^ mice fed a chronic HF diet was greater than the increase in food intake predicted^6^. While this was attributed to impaired adaptive thermogenesis^6^, an alternative/ complementary hypothesize could be that desacetyl-α-MSH and α-MSH produce the sexually dimorphic response to chronic HF diet by promoting gut energy extraction in female *Pomc*^tm1/tm1^ mice, which takes time to develop^6^.

There is strong evidence that melanocortin signaling pathway elements (i.e., MC4R and MC3R) are present in gut tissue. Hence gut structure and function are potentially influenced by melanocortin peptides^8,9^. In addition, there are potent anti-inflammatory functions of melanocortin hormones which may impact on gut structure and gut microbial dynamics^10,11^. Alpha-MSH suppresses the expression of genes for pro-inflammatory cytokines^12,13^; modulates T cell proliferation and stimulates the expression of IL-10 which is an anti-inflammatory cytokine^12^. Different dietary macronutrient compositions (e.g., HF or high-carbohydrate diets) trigger adaptive responses in gut anatomy to provide more efficacious nutrient metabolism^14^ which may be mediated by melanocortin peptides. Consistent with this, shorter and thicker microvilli, increased enterocyte counts, and increased nutrient absorption have been observed in rat intestines in response to a HF diet^15^.

The gut microbiome contributes to host energy regulation and homeostatic balance by: (1) modulating the host appetite and capability to extract dietary energy; (2) manipulating energy use and storage; and (3) influencing the host immune system^16–18^. The gut microbiota and their metabolites also influence intestinal structure by regulating intestinal stem cell proliferation and differentiation^19–21^. For example, butyrate inhibits intestinal self-repair by suppressing the proliferation of intestinal stem cells which results in smaller crypts and taller villi^19^. Since the immune system is tightly linked to the microbiome^22,23^, it is possible that reductions in the anti-inflammatory role of desacetyl-α-MSH and α-MSH in mice change the gut microbiota, microbial metabolites (short chain fatty acids [SCFAs]) and gut morphology in favor of weight gain.

Branched-chain amino acids (BCAAs), as nutritional signals, are involved in the gut-microbiome-brain axis to regulate the energy and metabolic balance (i.e., feeding behavior and insulin sensitivity)^24–26^. BCAAs are obtained from food, utilized by the intestinal cells, contribute to influence the gut microbiota composition and intestinal barrier health (reviewed in^26,27^). BCAAs pass through the blood brain barrier and activate the mammalian target of rapamycin (mTOR) in the hypothalamus^25^. mTOR is abundantly expressed on both POMC/CART and NPY/AgRP neurons, which act together to regulate feeding behavior^25^.

We hypothesize that desacetyl-α-MSH and α-MSH contribute to the regulation of gut morphology, microbiota composition, and the concentrations of SCFAs and BCAAs in mice on chronic HF diets. Male and female *Pomc*^tm1/tm1^ and WT mice were fed a HF or LF diet for 23 weeks from weaning^6^. Gut morphology, microbiota composition, concentrations of BCAAs and fecal SCFAs were analyzed and compared between treatment groups.

## Results

### *Pomc*^tm1/tm1^ mice have altered gut morphology

**Figure 1:**
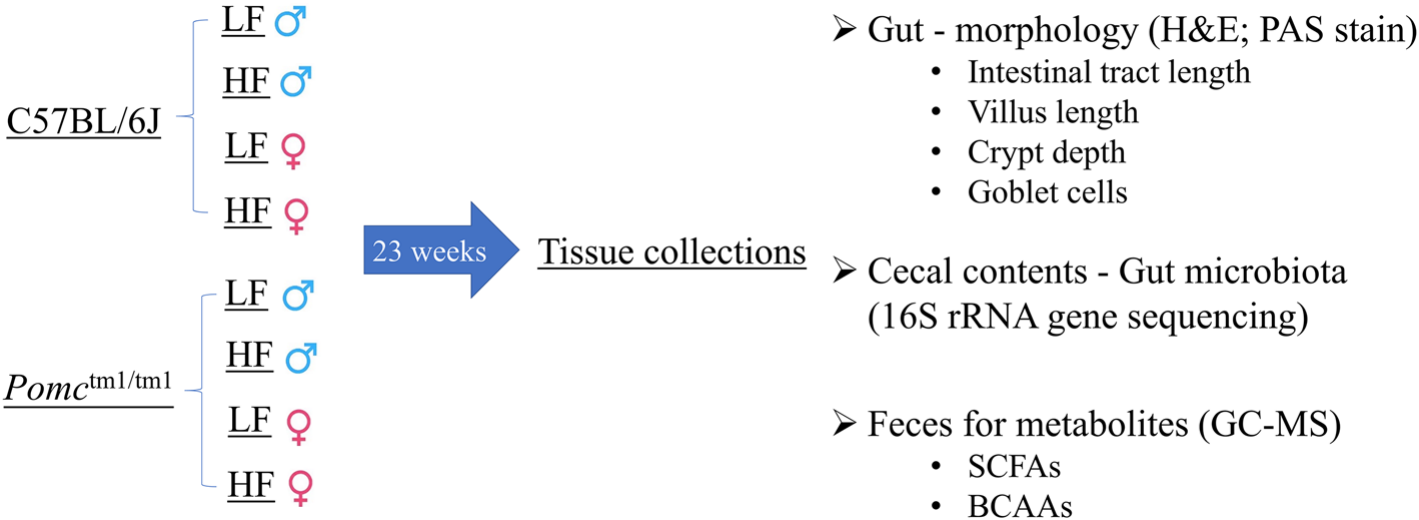
Cartoon of the study design. *Pomc*^tm^^1^^/tm1^ and C57BL/6J mice were assigned to eight groups according to the genotype, diet and sex differences ^6^. Diet was stratified according to kilocalories provided by the fat content (LF—fat = 10% of the kilocalories; HF—fat = 45% of kilocalories). Mice were culled at age 23 weeks for tissue collection. H&E: haematoxylin and eosin; PAS: periodic acid Schiff; SCFAs: short chain fatty acids; BCAAs: branched chain amino acids (BCAAs); GC–MS: gas chromatography mass spectrometry.

*Pomc*^tm1/tm1^ and C57BL/6J mice were assigned to eight groups according to the genotype, diet and sex differences^6^. Diet was stratified according to kilocalories provided by the fat content (LF—fat = 10% of the kilocalories; HF—fat = 45% of kilocalories). The groups were: male WT low fat (LF); male WT high fat (HF); male *Pomc*^tm1/tm1^ LF; male *Pomc*^tm1/tm1^ HF; female WT LF; female WT HF; female *Pomc*^tm1/tm1^ LF; and female *Pomc*^tm1/tm1^ HF^6^. Mice were kept in cages (4 mice cage^−1^, 2 cages group^−1^) and culled at age 23 weeks for tissue collection (Fig. 1).

**Figure 2:**
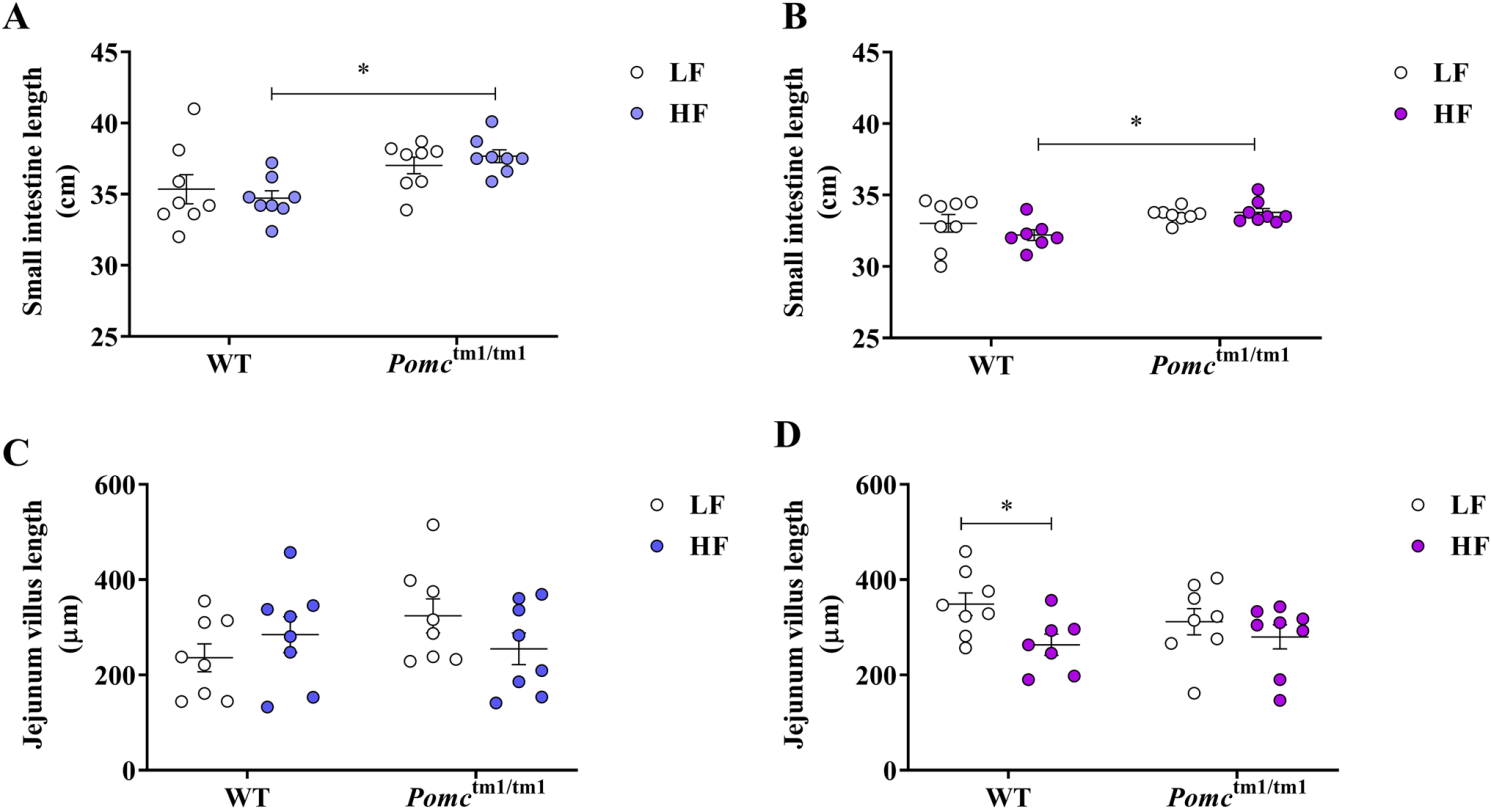
Small intestine and jejunum villus in male and female WT and *Pomc*^tm1/tm1^ mice fed on a LF or a HF diet. The small intestine and jejunum villus were measured in male (**A**, **C**) and female (**B**, **D**) mice fed on a LF or a HF diet for 23 weeks post weaning. Data are mean ± S.E.M. Significant differences were determined either using two-way ANOVA and Tukey’s post-hoc test or student’s *t* test (female jejunum villus length). *p < 0.05; WT: Wild-type; LF: Low-fat; HF: High-fat.

Colon length did not differ with diet or genotype in male or female mice (Tables S1, S2). Male and female *Pomc*^tm1/tm1^ mice showed a trend towards longer small intestines, when compared to WT mice. The observed difference in small intestine length was statistically significant in *Pomc*^tm1/tm1^ mice maintained on a chronic HF diet (Fig. 2A male: *p* = 0.0477 and B female: *p* = 0.0473) where it was proportional to increased body length, as observed previously^6^.

We measured villi length to determine if the trend towards increased small intestine length was associated with an increased absorption surface area. Diet was not associated with changes in villi length in male mice or female *Pomc*^tm1/tm1^ mice (Fig. 2C,D). By contrast, female WT mice had significantly shorter jejunum villi in response to a chronic HF diet (Fig. 2D, p = 0.0217).

Neither duodenum nor ileum showed genotype or diet specific differences in villus length or crypt depth, in either sex (Tables S1, S2). Furthermore, no group specific differences were observed in the numbers of goblet cells in the duodenum, jejunum or ileum (Tables S1, S2). These findings are consistent with a role for desacetyl-α-MSH and α-MSH in intestinal morphological adaptations that limit the nutrient absorptive surface in WT female mice chronically exposed to HF diets.

### *Pomc*^tm1/tm1^ genotype, chronic HF diet and sex were associated with gut microbiota composition

**Figure 3:**
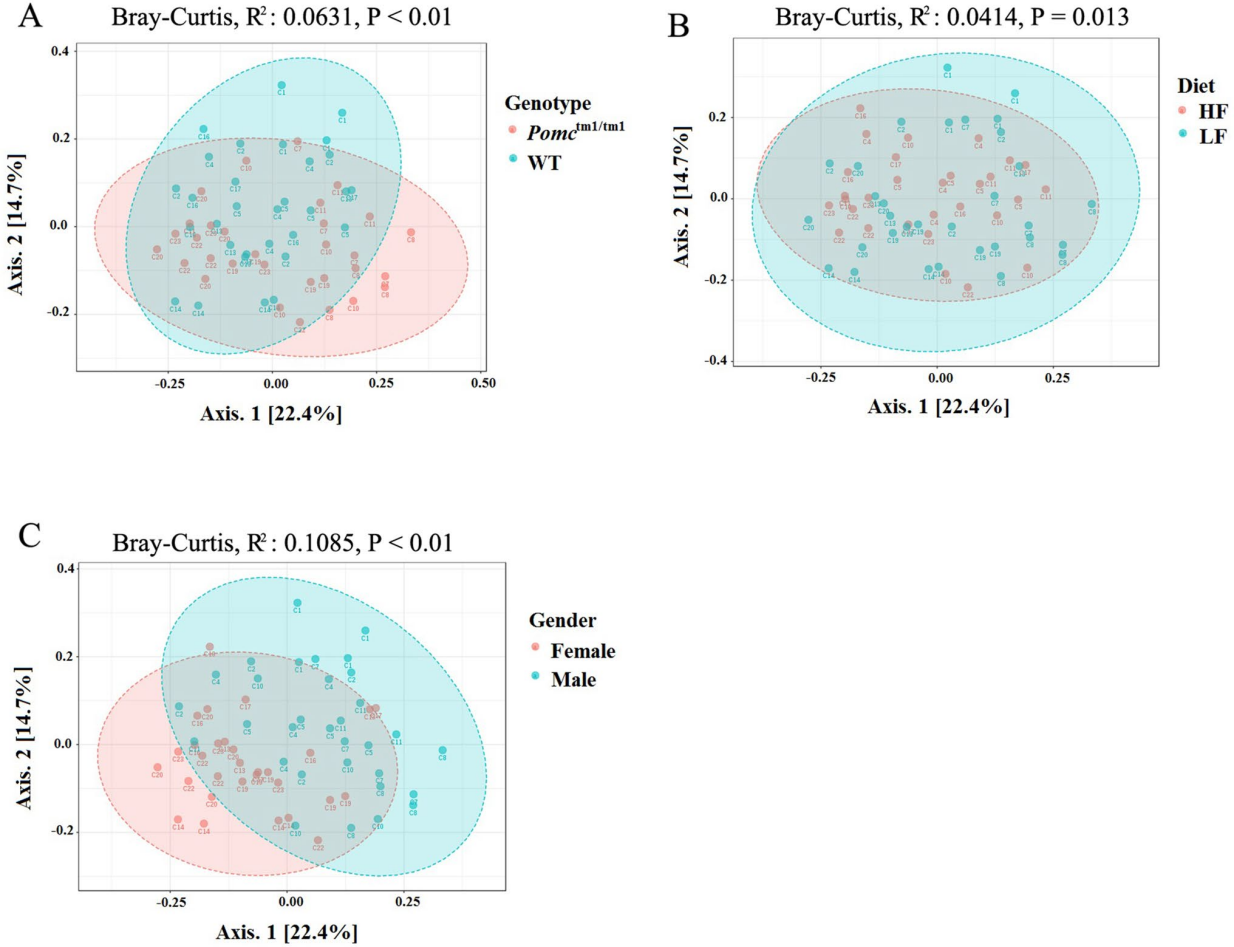
Principal coordinates analysis (PCoA) plots showing the β-diversity clustering patters of samples. (**A**) WT and *Pomc*^tm1/tm1^ mice; (**B**) mice fed on HF diet and LF diet; **C**, male and female mice. PCoA plots were based on Bray–Curtis dissimilarities. Cage ID was labelled. WT: wild type; LF: low fat; HF: high fat.

Cecal contents were collected to enable profiling of the microbial communities by 16S rRNA amplicon sequencing. The V3–V4 region of the 16S rRNA gene was amplified and paired-end reads (2 × 250 bp) were sequenced on an Illumina MiSeq. The numbers of quality-filtered sequences per sample ranged between 9899 and 61,808 (Table S4). In total, 5370 OTUs were identified using a 97% sequence similarity threshold. The impact of cage, maternal factor, genotype, diet or sex as single drivers of gut microbiota β-diversity was investigated using Permutational Analysis of Variance (PERMANOVA) (Fig. 3). We identified a significant cage effect in the mice microbiota (Bray–Curtis dissimilarity index, PERMANOVA: R^2^: 0.5284, *p* < 0.001). Therefore, cage-effect was corrected in all subsequent analyses. Maternal factor (i.e., litter) was not associated with differences in the Bray–Curtis dissimilarity index (PERMANOVA: *p* = 0.093).

Genotype and diet explained 6.3% and 4.1% of the observed variation in the gut microbiota, respectively (Fig. 3A,B). Sex explained 10.9% of the observed variation in the gut microbiota composition (Fig. 3C).

### Differences in gut microbiota between *Pomc*^tm1/tm1^ and WT mice were intensified in females on a chronic HF diet

Mice were stratified by diet, sex and genotype, and sequence reads rarefied to enable pairwise comparisons (Table S1A). Diet (HF v LF) was associated with gut microbiota composition in WT (Table 1; Fig. S1A, *p* = 0.01 and S2A, *p* = 0.004) and *Pomc*^tm1/tm1^ mice (Table 1; Fig. S1B, *p* = 0.013 and S1B*p* = 0.024), in both sexes. The *Pomc*^tm1/tm1^ genotype was associated with gut microbiota composition in female mice that were fed either LF or HF diets (Table 1; Fig. S1C, *p* = 0.003 and D, *p* = 0.001). In male mice, the *Pomc*^tm1/tm1^ genotype was significantly associated with gut microbiota only when the mice were fed a LF diet (Table 1; Fig. S1C, *p* = 0.001). The genotype effect in male mice was of borderline significance on the HF diet (Table 1; Fig. S1D, *p* = 0.045).

**Table 1:** Diet or genotype were associated with mouse cecal microbiota within genotype or diet, respectively.

Gender	Pairwise Comparison	Variant	Bray-Curtis Dissimilarity		Jaccard Index Jaccard Index	
			PERMANOVA		PERMANOVA	
			R^2^	*p *value	R^2^	*p *value
Male	WT LF versus WT HF	Diet	0.2467	0.001	0.2008	0.001
Male	*Pomc*^tm1/tm1^LF versus *Pomc*^tm1/tm1^HF	Diet	0.1692	0.013	0.1388	0.01
Male	WT LF versus *Pomc*^tm1/tm1^LF	Genotype	0.2569	0.001	0.2035	0.001
Male	WT HF versus *Pomc*^tm1/tm1^HF	Genotype	0.1163	0.045	0.1065	0.031
Female	WT LF versus WT HF	Diet	0.2306	0.004	0.1983	0.004
Female	*Pomc*^tm1/tm1^LF versus *Pomc*^tm1/tm1^HF	Diet	0.1545	0.024	0.1348	0.017
Female	WT LF versus *Pomc*^tm1/tm1^LF	Genotype	0.1868	0.003	0.1665	0.003
Female	WT HF versus *Pomc*^tm1/tm1^HF	Genotype	0.2855	0.001	0.2303	0.001

Interactions between diet and genotype explained less microbial variation in *Pomc*^tm1/tm1^ mice compared to WT mice (Bray–Curtis dissimilarity index, Table 1). The interaction between diet and genotype was sex-dependent. The *Pomc*^tm1/tm1^ genotype was associated with 26% of the variation in the male gut microbiota on the LF diet. However, this reduced to 12% of the variation in the gut microbiota when male *Pomc*^tm1/tm1^ mice were fed the HF diet (Bray–Curtis dissimilarity index, Table 1). By contrast, the genotype association with the gut microbiota in female *Pomc*^tm1/tm1^ mice increased from 19% on the LF diet to 29% on the HF diet (Bray–Curtis dissimilarity index, Table 1). These results suggest that there might be sex-dependent associations between diet, *Pomc*^tm1/tm1^ genotype and the gut microbiota.

### Exposure to chronic HF diet was only associated with reduced bacterial richness in WT mice

**Figure 4:**
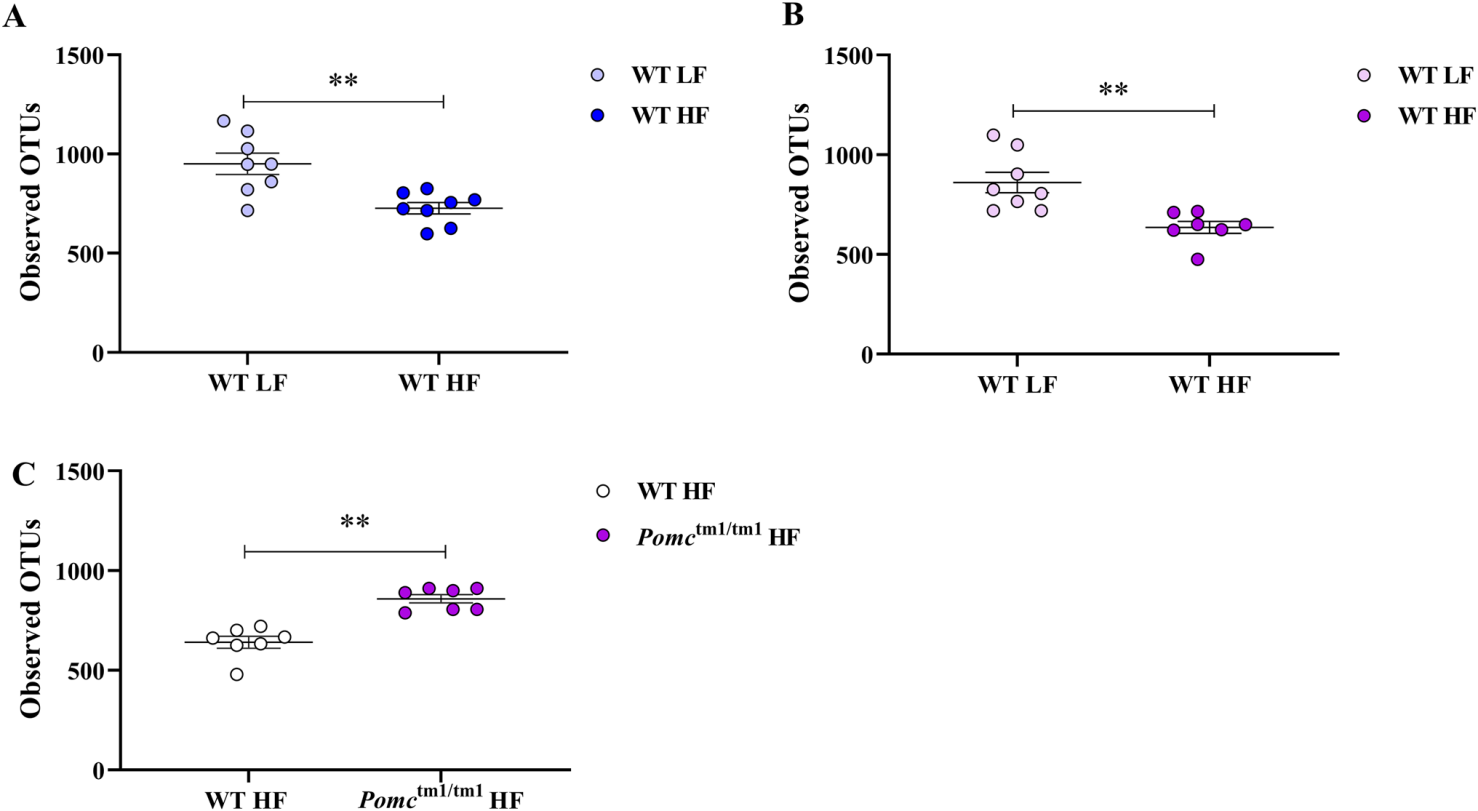
Microbial richness in male and female WT mice on either diet, and female *Pomc*^tm1/tm1^ mice on a HF diet. Alpha diversity was assessed using observed OTUs, n = 7 or 8 mice per group. Data are mean ± S.E.M. (**A**) male WT HF versus WT LF; (**B**) female WT HF versus WT LF; (**C**) female *Pomc*^tm1/tm1^ HF versus WT HF. The significant difference of the microbial richness between groups was tested by Mann–Whitney analysis, WT: Wild Type; LF: Low Fat; HF: High Fat.

We investigated genotype and dietary associations with α-diversity (i.e., number of observed OTUs per sample and the Shannon’s diversity index). Eight pairwise comparisons were performed to test either genotype effect within diet and sex on bacterial α-diversity, or diet effect within genotype and sex on bacterial α-diversity (Table 2). HF diet was significantly associated with reduced bacterial richness in male and female WT (*p* = 0.0086 and B, *p* = 0.0014) but not *Pomc*^tm1/tm1^ mice (Table 2). When investigating the *Pomc*^tm1/tm1^ genotype effect, *Pomc*^tm1/tm1^ females on HF showed significantly higher bacterial richness than WT females on the chronic HF diet (Fig. 4C, *p* = 0.0021). However, no significant differences were observed in the other pairwise comparisons (Table 2). Analyses of the Shannon diversity index identified no significant differences between diet, or genotype (Table 2).

**Table 2:** Diet and genotype were associate with the α-diversity of mouse cecal microbiota.

Gender	Comparison	Variant	Observed OTUs Index	Shannon Index
			Mann-Whitney *p* value	Mann-Whitney *p *value
Male	WT LF versus WT HF	Diet	0.01	0.7985
Male	*Pomc*^tm1/tm1^LF versus *Pomc*^tm1/tm1^HF	Diet	0.5054	0.6454
Male	WT LF versus *Pomc*^tm1/tm1^ LF	Genotype	0.1949	0.9591
Male	WT HF versus *Pomc*^tm1/tm1^ HF	Genotype	0.9591	0.7985
Female	WT LF versus WT HF	Diet	0.001	0.1206
Female	*Pomc*^tm1/tm1^ LF versus *Pomc*^tm1/tm1^ HF	Diet	0.6022	0.2319
Female	WT LF versus *Pomc*^tm1/tm1^ LF	Genotype	0.7928	0.7985
Female	WT HF versus *Pomc*^tm1/tm1^ HF	Genotype	0.01	0.5350

### Sex-specific alterations of acetic and propionic acid concentrations were observed between genotypes

More than 90% of the gut microbiota in WT and *Pomc*^tm1/tm1^ mice was composed of *Bacteroidetes*, *Firmicutes* and *Proteobacteria* (Fig. S3). Linear modelling (MaAsLin) did not identify significant associations between genotype, diet, SCFAs, BCAAs and specific bacterial taxa at phylum or genus levels within sex.

**Figure 5:**
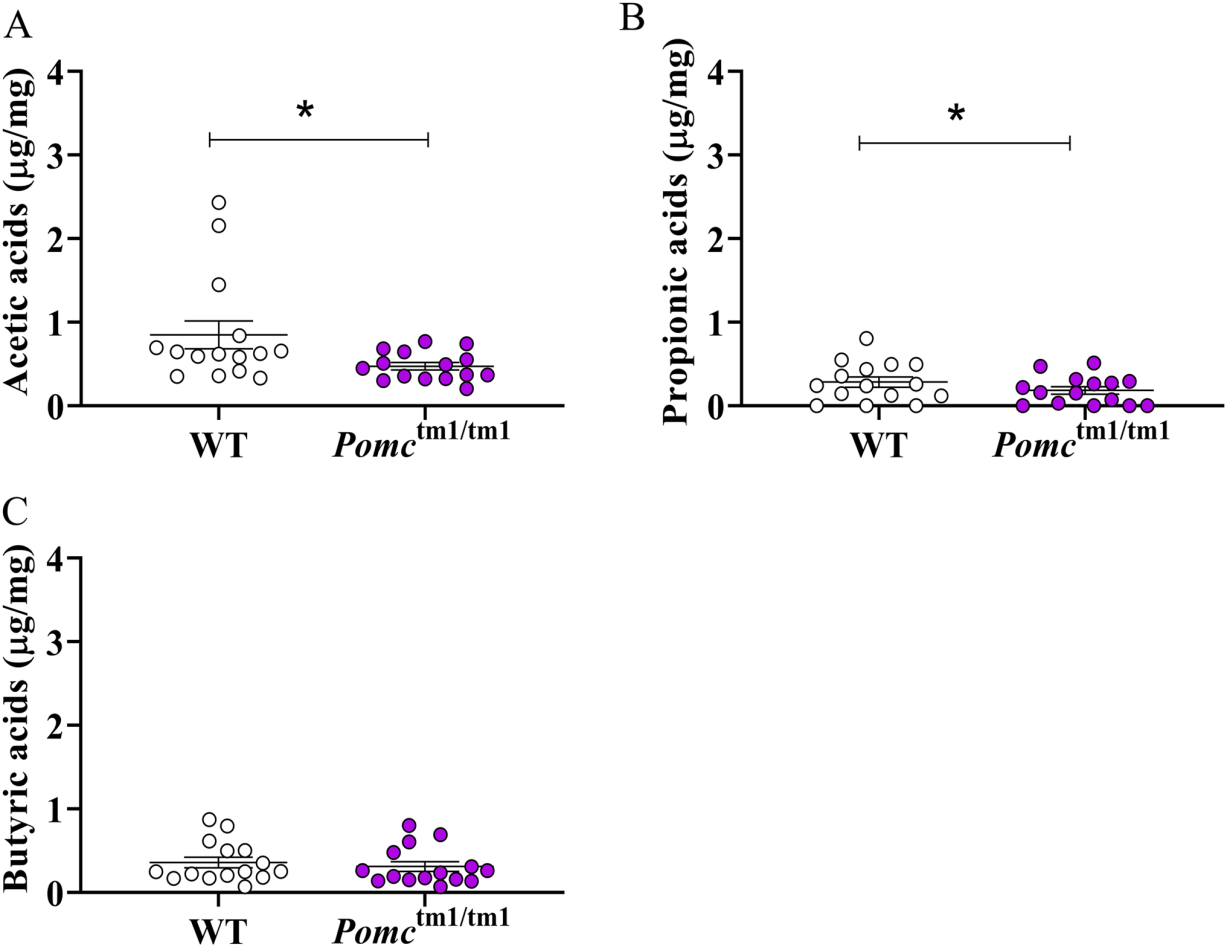
Fecal SCFAs concentrations in females. SCFAs were measured using PCF/GC–MS method, and the final concentrations were normalized by dried feces weights; (**A**) acetic acids; (**B**) propionic acid; (**C**) butyric acid; Data are mean ± S.E.M. The differences were determined by using linear mixed-effects models, *p < 0.05.

Linear mixed-effect models identified significantly gender-specific effect of genotypes on the fecal acetic acid (gender: genotype: *p* = 0.0314) and propionic acid (gender: genotype: *p* = 0.0129). Specifically, WT females had 2.78 times the concentration of acetic acid (*p* = 0.0239, Fig. 5A), and 3.34 times the concentration of propionic acid (*p* = 0.0099, Fig. 5B) observed in *Pomc*^tm1/tm1^ females. By contrast, there were no significant differences in acetic and propionic acid levels between male genotypes (Fig. S4A and B). No significant interactions or main effects were observed for butyric acid (Figs. 5C, S1C) or BCAA concentrations (Fig. S5).

## Discussion

We have identified anatomical changes (e.g., increased intestinal length) in male and female *Pomc*^tm1/tm1^ mice when they were fed a chronic HF diet. Deletion of desacetyl-α-MSH and α-MSH from the female mice resulted in loss of protection from diet induced obesity and a failure to reduce villus length on exposure to the chronic HF diet. These anatomical changes were accompanied by changes in the gut microbiota and fecal SCFA concentrations. We conclude that these structural changes to the gut contributed to the observed increase in female body weight that occurred in the absence of an appreciable increase in food intake or caloric consumption^6^.

It has previously been demonstrated that adult male mice fed a HF diet (45% kcal from fat) for 12 weeks had an enlarged villus area in the small intestine^28^. Similarly, short term exposure to HF diets (45% kcal from fat; 20 weeks) increase villus height in the jejunum of adult male and female Sox9-EGFP reporter mice^29^. We contend that the differences between these studies and ours, despite the use of the same diet, stem from exposure of our animals to a chronic HF diet from weaning. Therefore, the age of exposure might be critical for structural adaptations of the intestine that determine the absorptive surface area available for nutrient absorption. Human studies of total small bowel length show a remarkable variation from 2.5 to 13 m. It is generally longer in males than females^30^. A weak correlation with height has been reported although there is no correlation with degree of obesity in abdominal surgery cohorts^30^.

Org et al. observed gender-specific microbiota compositions across 86 strains^31^. For example, there were more phylum *Actinobacteria* and *Tenericutes* in males; while genus *Dorea, Coprococcus* and *Ruminococcus* were more abundant in females^31^. The gender specific gut microbiota composition Org et al. observed was genotype-dependent when strains were analyzed individually^31^. The most apparent sex specific differences in gut microbiota among the 86 strains were observed in C57BL/6J mice; and the gender specific microbiota composition in C57BL/6J mice is greater in response to a HF diet^31^. Sexually dimorphic changes in the gut microbiome are likely mediated by sex-dependent immune differences^32^. POMC-derived peptides are key regulators of innate and adaptive immunity^33^. Consistent with this, our results identified that deletion of desacetyl-α-MSH and α-MSH is associated with sex-specific differences in the gut microbiota, in response to exposure to a chronic HF diet.

The association between reduced bacterial diversity and obesity has been described by some^16,34^, but not all studies^35^. Similarly, inconsistent results have been observed between bacterial richness and obesity in mouse studies^29,36^. In this study, we observed that HF compared with LF diet was associated with reduced bacterial richness in male and female WT mice, but not in male or female *Pomc*^tm1/tm1^ mice. This reduction in bacterial richness was, therefore, independent of sex and independent of obesity, since the HF diet-induced obesity in male but not female WT mice and the HF diet exacerbated both male and female *Pomc*^tm1/tm1^ obesity. Interestingly, an increased bacterial richness was observed in female *Pomc*^tm1/tm1^ mice compared to WT mice on a HF diet, while this *Pomc*^tm1/tm1^ genotype effect was absent in female mice on a LF diet or male *Pomc*^tm1/tm1^ mice regardless of diet. This is consistent with the results of the β-diversity analysis, which showed the effect of *Pomc*^tm1/tm1^ genotype on gut microbiota composition was intensified by a HF diet, but not in males. Taken together, our study indicates a sex-specific interaction between melanocortin peptides and diet in influencing gut microbiome. The most likely implication of these findings are that α-MSH acts to protect female mice from chronic HFD related obesity, and in the absence of these peptides, there are alterations to the gut microbiota which promote increased energy extraction from the HFD. However, there are other potential explanations that need to be considered.

We cannot conclude a causal relationship between the gut microbiota, *Pomc*^tm1/tm1^ genotype, HF diet, and fecal SCFAs. *Pomc*^tm1/tm1^ mice gained significantly more body weight than WT mice on either the LF or chronic HF diets^5,6^. Given that food consumption influences gut microbiota composition^37,38^, our experimental design does not allow us to determine if the significant difference in body weight between groups was causal in changing the gut microbiota composition and fecal SCFA concentrations. For example, HF diets are known to induce hyperphagia by reducing sensitivity to satiety signals (e.g., cholecystokinin) in mice^39^. HF diet also induces inflammation in the hypothalamus, which has impacts on appetite-stimulating NPY/AgRP neurons^40^. Therefore, it remains possible that the altered gut microbiota composition we observed is a consequence of: (1) hyperphagia induced by desacetyl-α-MSH and α-MSH deficiency; or (2) the chronic HF diet itself. Pair-fed mice would be a better control, than *ad-libitum* fed mice, to untangle this effect. Since the female *Pomc*^tm1/tm1^ mice gained more body weight than WT females when fed a LF diet, despite consuming the same amount of this food^6^, it is likely that the differences between these groups are due to the deficiency of the desacetyl-α-MSH and α-MSH impacting on increased energy extraction from the diet, although decreased energy expenditure could also be involved.

Female *Pomc*^tm1/tm1^ mice had lower fecal acetic acid and propionic acid levels, regardless of diet. The main source of SCFAs in this study was dietary fiber. The fiber concentration of LF diet (50 g/1055.05 g) was lower than the HF diet 50 g/858.15 g). However, the diet effect on SCFAs was not significantly different and could be masked by genotype and sex effects. Acetic acid, butyric acid and propionic acid account for > 95% of SCFAs, and each compound has different distributions and individual functions^41^. For example, butyric acids are mainly produced by members of the phylum *Firmicutes* (e.g., *Eubacterium*, and *Roseburia* species) and used by the colon epithelial cells^42^. By contrast, acetic acid and propionic acid can pass through the blood brain barrier and are known to contribute to the regulation of lipid metabolism, insulin sensitivity, anti-inflammation and feeding behavior^41,43^. The reduced acetic acid and propionic acid levels we measured are consistent with the obese phenotype in female *Pomc*^tm1/tm1^ mice, based on other studies (reviewed in^44^). This study provides further evidence supporting the existence of sex-specific differences in gut communities and metabolites in response to HF diet.

Fecal SCFA concentrations need not mirror concentrations of SCFAs in the peripheral circulation. SCFAs are the end products of anaerobic metabolism in the gut and generally have the highest concentrations in the large intestine^45^. After local use by the colonocytes, SCFAs are released into the portal, hepatic and peripheral venous blood^45^. Therefore, we are unable to conclude whether the reduced fecal SCFAs in female *Pomc*^tm1/tm1^ mice were due to decreased production or an increase in circulating SCFAs in these mice. Future analyses involving plasma SPME headspace GCMS may help to shed light on this.

The results of our study are consistent with the POMC derived peptides having a sex-specific role in modulating energy balance beyond appetite alone^6^. We have shown that the sex-specific effect of POMC peptides is associated with altered gut morphology and gut microbiota, which could be either a consequence or a cause of the *Pomc*^tm1/tm1^ obesity, or both, given that there is bidirectional communication between the gut microbiome and brain. Cross-talk between the gut and brain is essential for integrating peripheral and central immune, metabolic and endocrine signals to maintain energy and metabolism homeostasis^46–48^. It remains possible that the microbes contribute to this gut–brain communication by: (1) the production and activation of appetite-regulating hormones (e.g., cholecystokinin)^49^; (2) impacts on central appetite regulation through disruption of the blood–brain barrier (BBB)^50,51^, or (3) production of peptides that mimic appetite-regulating hormones (e.g., C1pB, as a mimetic of α-MSH)^52^. Resolving this will require fecal microbiome transfer experiments to establish a causal relationship for the microbiome in *Pomc*^tm1/tm1^ mediated obesity.

In conclusion, this study supports a role for POMC derived peptides in conferring protection from chronic HF diet related obesity in females through compensatory changes in the gut morphology and gut microbiota that collectively limit nutrient absorption. We also demonstrated a sex-specific effect of *Pomc*^tm1/tm1^ genotype in influencing gut microbiota in the presence of a HF diet.

## Methods

### Animals

The mice used in this study were previously described^6^. All experimental procedures involving mice were approved by the Auckland University Animal Ethics Committee (Approval number 001133) and conformed to the Animal Welfare Act 1999. Briefly, mice were assigned to eight groups based on diet, genotype and sex differences; male WT low fat (LF), male WT high fat (HF), male *Pomc*^tm1/tm1^ LF, male *Pomc*^tm1/tm1^ HF, female WT LF, female WT HF, female *Pomc*^tm1/tm1^ LF and female *Pomc*^tm1/tm1^ HF^6^. Fat accounted for 10% of the kilocalories in the LF diet (Research diets, Inc. D12450B, New Brunswick, NJ, USA;^6^). By contrast, fat accounted for 45% of kilocalories in the HF diet (Research diets, Inc. D12451, New Brunswick, NJ, USA). Mice for each group were randomized into two cages after weaning, such that each cage contained four mice derived from different mothers^6^. Totally, 63 mice were sacrificed for analysis at age 23 weeks (32 male mice; 31 female mice: one mouse died on the way). There were no more than three mice per group from the same mother (Table S4;^6^).

### Tissue collection

Mice were culled by CO_2_ asphyxiation for tissue collection after 23 weeks on a LF or HF diet^6^. The intestinal tract, from the duodenum to colon, was collected immediately post cull. Intestinal length was measured using a ruler. Ice-cold phosphate buffered saline was flushed through the gut tissues using a BD PrecisionGlide Needle (18G 1 ½ TW 1.2 × 38 mm). The small intestine was dissected into the duodenum, jejunum, and ileum. Segments (2 cm) of the duodenum, jejunum and ileum were collected for histological analysis.

The caecum was collected for gut microbiota analysis. The contents of the caecum were transferred to RNA- and DNA-free Eppendorf tubes, using sterile forceps. Samples were snap frozen on dry ice. Fecal pellets, for quantification of SCFAs and BCAAs, were collected between 9 am and 12 pm, 1–7 days before the mice were culled. Pellets were stored frozen (-80 °C).

### Histology

All histological images were acquired using a Zeiss AxioCam HRc Axioskop 2 mot *plus* microscope. Morphometric analysis and cell counts were performed using Image J 1.46r^53^.

Intestinal segments from the duodenum, proximal jejunum, and distal ileum were fixed in 10% neutral buffered formalin for 24 h, before storage in 70% ethanol. Tissues were embedded longitudinally in paraffin using standard techniques. Tissues were sectioned (5 µm cross sections). Four serial cross sections, from each animal, were obtained and mounted on each slide (n = 8 or 7 mice per group). One slide per tissue per animal was used for villus length and crypt depth measurement. One slide was used for goblet cell analysis.

Haematoxylin and eosin (H&E) staining was used to evaluate villus length and crypt depth^54,55^. All well-oriented villi and crypts from four serial sections per tissue, per mouse, were used to determine villus height and crypt depth. The villus length was defined as the length between the top to base of each villus, excluding the crypt, using 20 × magnification. The crypt depth was defined as the distance from the base of the villus to the crypt interface with the outer muscularis mucosae^56^.

Periodic Acid Schiff (PAS) stain was used to visualize goblet cells^57^. Goblet cell numbers in each section were counted for all well-oriented villi. The number of goblet cells was normalized to the area of each villus, measured using Image J 1.46r^53^.

### DNA extraction

DNA was extracted using the QIAamp Fast DNA Stool Mini Kit (Qiagen). Frozen cecal samples (~ 60 mg) were lysed in LoBind Eppendorf tubes using a TissueLyser II (10 min, 30 Hz) following addition of 1 mL glass beads (acid-washed, 106 µm [-140 U.S. sieve], Sigma-Aldrich) and lysis buffer (InhibitEX buffer and proteinase K, Qiagen). DNA concentration was measured with the Qubit Quant-iT dsDNA High Sensitivity assay kit (Thermo Fisher Scientific Inc.). DNA quality was determined with a NanoDrop 1000 spectrophotometer (Thermo Fisher Scientific Inc.) and by visual inspection following electrophoresis (1.2% (w/v) agarose gel in Tris–acetate-EDTA buffer).

### 16S ribosomal RNA (16S rRNA) amplicon library preparation

DNA samples were stored (4 °C) before being sent for 16S rRNA gene amplicon sequencing (New Zealand Genomics Laboratory; NZGL). Sequencing libraries were prepared for the Illumina Miseq system using the 16S rRNA Metagenomic Sequencing Library Preparation protocol (Illumina). The 16S rRNA V3-V4 region (460 bp) was amplified from DNA (12.5 ng) in a 25 μl reaction, containing 5 μl of 1 μM 16S Amplicon Forward Primer (5′-TCGTCGGCAGCGTCAGATGTGTATAAGAGACAGCCTACGGGNGGCWGCAG-3′), 5 μl of 1 μM 16S Amplicon polymerase chain reaction (PCR) Reverses Primer (5′-GTCTCGTGGGCTCGGAGATGTGTATAAGAGACAGGACTACHVGGGTATCTAATCC-3′), and 12.5 μl of 2 × KAPA HiFi HotStart Ready Mix. The PCR program was (95 °C for 3 min; 25 cycles [95 °C for 30 s, 55 °C for 30 s, 72 °C for 30 s]; and a final extension [72 °C for 5 min]). Illumina adapter overhang nucleotide sequences were added, amplicons purified (AMPure XP beads; 20 μl per amplicon), before dual indices and Illumina sequencing adapters were attached (NextEra XT Index Kit). Amplicons were purified (AMPure XP beads) and aliquots (1 μl of a 1:50 dilution) were quality checked using a Bioanalyser DNA 1000 chip (Agilent Technologies).

16S gene amplicon sequencing libraries were quantified using a Qubit Fluorometer. Libraries were normalized, pooled, denatured (0.1 M NaOH) and diluted to 20 pM. PhiX (PhiX Control Kit v3; Illumina) was added (5% final concentration). PhiX spiked libraries were pooled (600 μl) and loaded onto an Illumina MiSeq according to the manufacturer’s instructions (MiSeq reagent Kit v2). FastQ files (2 × 250 bp Paired-End reads) were generated, run quality checked using the PhiX Control and each paired-end sequence assigned to its sample using the multiplexing index. Paired-end reads were quality filtered and assembled using Paired-End read mergeR (version 0.9.10).

### Analysis of 16S rRNA gene amplicon sequences

Quality filtered and assembled sequences were analyzed using Quantitative Insights Into Microbial Ecology (QIIME, version 1.9.1;^58^). Raw MiSeq sequencing data were mapped against the PhiX genome using Bowtie2 (https://bowtie-bio.sourceforge.net/bowtie2/index.shtml) and the PhiX sequences removed. Adapter sequences were then removed (fastq-mcf” programme ea-utils suite of tools [https://code.google.com/p/ea-utils/; version 1.1.2–621]). DynamicTrim was used to trim sequences to their longest contiguous segment for which quality scores were < 0.01 (SolexaQA++ software; https://solexaqa.sourceforge.net/). Quality filtered sequences were reformatted using QIIME (version 1.9.1) for 16S metagenomic processing.

The open-reference operational taxonomic units (OTUs) picking method was used to identify bacteria and compute their distribution across different taxonomic levels. Specifically, reads were clustered into OTUs using the UCLUST algorithm and a 97% sequence similarity threshold. Singleton OTUs were removed. Taxonomic annotation of the cluster centroids was performed using the Green genes 16S gene database (GG, version 13_8)^59,60^. Rarefaction curves based on the phylogenetic diversity (PD) metric, showed a trend towards saturation and confirmed that the sequence depth in each sample was sufficient to include most OTUs (Fig. S6).

β-diversity analyses were performed in MicrobiomeAnalyst^61^ using the Jaccard and Bray–Curtis dissimilarities matrix and visualized by Principal Coordinates Analysis (PCoA). The Jaccard index compares microbiome profiles in terms of presence/absence of OTUs^62^. By contrast, the Bray–Curtis dissimilarity index compares OTU abundance profiles^63^.

PERMANOVA was used to test for significant differences in gut microbial communities and structure between groups. PERMANOVA, with 999 permutations, returns a P value for statistical significance, alongside an R^2^ value, indicative of the proportion of variation explained by a specific grouping variable.

The Shannon diversity index (accounting for both abundance and evenness) and richness (the number of observed OTUs) was used to measure α-diversity of the bacterial communities. The Mann–Whitney *U* test was used to identify any significant differences between LF and HF diets, and between WT and *Pomc*^tm1/tm1^ genotypes in terms of bacterial richness and evenness. Cage effects were not accounted for in this test.

### Fecal SCFA and BCAA quantification

SCFAs and Branched Chain Amino Acids (BCAAs) were analyzed using propyl chloroformate (PCF) derivatization followed by gas chromatography mass spectrometry (GC–MS)^64^. In brief, fecal samples were mixed with NaOH (0.005 M) containing stable-isotope labelled internal standards (acetic acid-d4, l-leucine-d10 and l-alanine-d4, all at a concentration of 25 μg/ ml). A mixture of 1-propanol: pyridine (3:2, v/v) and propyl chloroformate were added and the derivatives were extracted with hexane. The upper hexane layer was transferred to a GC–MS vial for analysis. The remaining solid fecal matter was placed in a centrifugal vacuum concentrator (Thermo SpeedVac) until visibly dry (~ 5 h). The dried fecal biomass (Tables S5, S6) was used for normalization.

SCFAs and BCAAs were separated and identified using an Agilent 7890A GC coupled to an Agilent 5975C MSD. Quantification was performed using a 10-point calibration curve (0.5–250 µg/ml) for each target analyte, normalizing by internal standard and using the regression function of the curve to predict the concentration for any given peak area. Raw GC–MS data were converted to NetCDF files using MSD ChemStation G1701FA F.01.01.2317. MassOmics (XCMS R packaged in GUI) was used to integrate peak area.

### Multivariate association testing

Individual associations between bacterial genera or phyla and genotype, diet, SCFAs and BCAAs were analyzed by linear modelling in multivariate association with linear model (MaAsLin)^65^. Cage effect was accounted for when testing the differences of SCFAs and BCAAs between genotypes, diets and genders using linear mixed-effects models (nlme, version 3.1–137)^66^.

Linear mixed-effects models (nlme, version 3.1–137, R version 3.5.0)^66^ were used to test for differences in SCFA and BCAA concentrations between genotypes, diets and gender. Zero values were removed, and the data were logarithm transformed to satisfy normality assumption, prior to analysis. Cage effect was a confounder in this study and was accounted for in the linear mixed-effect models. Six linear mixed-effects models were generated based on six response variables (acetic acid, butyric acid, propionic acid, l-leucine, l-isoleucine and l-valine) with diet, genotype and gender modelled as the fixed effects and cage modelled as random effects. Analysis of variance (ANOVA) was used to test for the three-way interactions, two-way interactions and main effects. Pairwise comparisons that passed the *p* < 0.05 threshold of significance in the ANOVA were subjected to post-hoc analysis.

## Supplementary information

Supplementary Information

## Data Availability

All sequences are available through the Short Read Archive: Bioproject PRJNA526611.

## References

[1] Butler AA (2006). The melanocortin system and energy balance. Peptides.

[2] Hill JW, Faulkner LD (2017). The role of the melanocortin system in metabolic disease: New developments and advances. Neuroendocrinology.

[3] Horvath TL (2010). Synaptic input organization of the melanocortin system predicts diet-induced hypothalamic reactive gliosis and obesity. Proc. Natl. Acad. Sci. USA.

[4] Cone RD (2005). Anatomy and regulation of the central melanocortin system. Nat. Neurosci..

[5] Mountjoy KG (2018). Desacetyl-alpha-melanocyte stimulating hormone and alpha-melanocyte stimulating hormone are required to regulate energy balance. Mol. Metab..

[6] Hubbard K (2019). Chronic high-fat diet exacerbates sexually dimorphic *Pomc*^tm1/tm1^ mouse obesity. Endocrinology.

[7] Blaak E (2001). Gender differences in fat metabolism. Curr. Opin. Clin. Nutr. Metab. Care.

[8] Gantz I (1993). Molecular cloning of a novel melanocortin receptor. J. Biol. Chem..

[9] Panaro BL (2014). The melanocortin-4 receptor is expressed in enteroendocrine L cells and regulates the release of peptide YY and glucagon-Like peptide 1 in vivo. Cell Metab..

[10] Singh M, Mukhopadhyay K (2014). Alpha-melanocyte stimulating hormone: An emerging anti-inflammatory antimicrobial peptide. Biomed. Res. Int..

[11] Rajora N (1997). alpha-MSH modulates experimental inflammatory bowel disease. Peptides.

[12] Cooper A (2005). Alpha-melanocyte-stimulating hormone suppresses antigen-induced lymphocyte proliferation in humans independently of melanocortin 1 receptor gene status. J. Immunol..

[13] Manna SK, Aggarwal BB (1998). Alpha-melanocyte-stimulating hormone inhibits the nuclear transcription factor NF-kappa B activation induced by various inflammatory agents. J. Immunol..

[14] Covasa M (2010). Deficits in gastrointestinal responses controlling food intake and body weight. Am. J. Physiol. Regul. Integr. Compar. Physiol..

[15] Goda T, Takase S (1994). Effect of dietary fat content on microvillus in rat jejunum. J. Nutr. Sci. Vitaminol..

[16] Turnbaugh PJ (2008). A core gut microbiome in obese and lean twins. Nature.

[17] Ley RE (2010). Obesity and the human microbiome. Curr. Opin. Gastroenterol..

[18] Bäckhed F (2004). The gut microbiota as an environmental factor that regulates fat storage. Proc. Natl. Acad. Sci..

[19] Kaiko GE (2016). The colonic crypt protects stem cells from microbiota-derived metabolites. Cell.

[20] Sommer F (2015). Site-specific programming of the host epithelial transcriptome by the gut microbiota. Genome Biol..

[21] Shanahan MT, Tanabe H, Ouellette AJ (2011). Strain-specific polymorphisms in Paneth cell alpha-defensins of C57BL/6 mice and evidence of vestigial myeloid alpha-defensin pseudogenes. Infect. Immunol..

[22] Vijay-Kumar M (2010). Metabolic syndrome and altered gut microbiota in mice lacking Toll-like receptor 5. Science (New York, N.Y.).

[23] Hooper LV, Littman DR, Macpherson AJ (2012). Interactions between the microbiota and the immune system. Science (New York N.Y.).

[24] Yoon MS (2016). The emerging role of branched-chain amino acids in insulin resistance and metabolism. Nutrients.

[25] Cota D (2006). Hypothalamic mTOR signaling regulates food intake. Science.

[26] Zhou H (2018). Regulation of intestinal health by branched-chain amino acids. Anim. Sci. J..

[27] Ma N, Ma X (2019). Dietary amino acids and the gut-microbiome-immune axis: Physiological metabolism and therapeutic prospects. Compr. Rev. Food Sci. Food Saf..

[28] Campbell SC (2016). The effect of diet and exercise on intestinal integrity and microbial diversity in mice. PLoS ONE.

[29] Cox LM (2014). Altering the intestinal microbiota during a critical developmental window has lasting metabolic consequences. Cell.

[30] Bekheit M (2020). Correlation between the total small bowel length and anthropometric measures in living humans: Cross-sectional study. Obes. Surg..

[31] Org E (2016). Sex differences and hormonal effects on gut microbiota composition in mice. Gut. Microbes.

[32] Fransen F (2017). The impact of gut microbiota on gender-specific differences in immunity. Front. Immunol..

[33] Taylor AW, Lee DJ (2011). The alpha-melanocyte stimulating hormone induces conversion of effector T cells into treg cells. J. Transpl..

[34] Turnbaugh PJ (2008). Diet-induced obesity is linked to marked but reversible alterations in the mouse distal gut microbiome. Cell Host Microbe.

[35] Sze, M. A. & Schloss, P. D. Looking for a signal in the noise: Revisiting obesity and the microbiome. *mBio.* **7**(4), e01018–16. 10.1128/mBio.01018-16 (2016). Erratum in: *MBio*. **8**(6) (2017). 10.1128/mBio.01018-16 PMC4999546 27555308

[36] Xiao L (2017). High-fat feeding rather than obesity drives taxonomical and functional changes in the gut microbiota in mice. Microbiome.

[37] Scott KP (2013). The influence of diet on the gut microbiota. Pharmacol. Res..

[38] Zhang C (2013). Structural modulation of gut microbiota in life-long calorie-restricted mice. Nat. Commun..

[39] Savastano DM, Covasa M (2005). Adaptation to a high-fat diet leads to hyperphagia and diminished sensitivity to cholecystokinin in rats. J. Nutr..

[40] Dalvi PS (2017). High fat induces acute and chronic inflammation in the hypothalamus: Effect of high-fat diet, palmitate and TNF-alpha on appetite-regulating NPY neurons. Int. J. Obes. (Lond.).

[41] Louis P, Hold GL, Flint HJ (2014). The gut microbiota, bacterial metabolites and colorectal cancer. Nat. Rev. Microbiol..

[42] Riviere A (2016). Bifidobacteria and butyrate-producing colon bacteria: Importance and strategies for their stimulation in the human gut. Front. Microbiol..

[43] Parker A, Fonseca S, Carding SR (2020). Gut microbes and metabolites as modulators of blood-brain barrier integrity and brain health. Gut Microbes.

[44] Byrne CS (2015). The role of short chain fatty acids in appetite regulation and energy homeostasis. Int. J. Obes. (Lond.).

[45] den Besten G (2013). The role of short-chain fatty acids in the interplay between diet, gut microbiota, and host energy metabolism. J. Lipid Res..

[46] Borre YE (2014). Microbiota and neurodevelopmental windows: Implications for brain disorders. Trends Mol. Med..

[47] Burokas A (2015). Microbiota regulation of the Mammalian gut-brain axis. Adv. Appl. Microbiol..

[48] Agusti A (2018). Interplay between the gut-brain axis, obesity and cognitive function. Front. Neurosci..

[49] Raybould HE (2010). Gut chemosensing: Interactions between gut endocrine cells and visceral afferents. Auton. Neurosci..

[50] Banks WA (2015). Lipopolysaccharide-induced blood-brain barrier disruption: Roles of cyclooxygenase, oxidative stress, neuroinflammation, and elements of the neurovascular unit. J. Neuroinflamm..

[51] von Meyenburg C (2004). Role for CD14, TLR2, and TLR4 in bacterial product-induced anorexia. Am. J. Physiol. Regul. Integr. Compar. Physiol..

[52] Tennoune N (2014). Bacterial ClpB heat-shock protein, an antigen-mimetic of the anorexigenic peptide alpha-MSH, at the origin of eating disorders. Transl. Psychiatry.

[53] Schneider CA, Rasband WS, Eliceiri KW (2012). NIH Image to ImageJ: 25 years of image analysis. Nat. Methods.

[54] Dekaney CM (2007). Expansion of intestinal stem cells associated with long-term adaptation following ileocecal resection in mice. Am. J. Physiol. Gastrointest Liver Physiol..

[55] Dehmer JJ (2011). Expansion of intestinal epithelial stem cells during murine development. PLoS ONE.

[56] Radtke F, Clevers H (2005). Self-renewal and cancer of the gut: Two sides of a coin. Science.

[57] Yamabayashi S (1987). Periodic acid—Schiff—Alcian Blue: A method for the differential staining of glycoproteins. Histochem. J..

[58] Caporaso JG (2010). QIIME allows analysis of high-throughput community sequencing data. Nat. Methods.

[59] Edgar RC (2010). Search and clustering orders of magnitude faster than BLAST. Bioinformatics.

[60] DeSantis TZ (2006). Greengenes, a chimera-checked 16S rRNA gene database and workbench compatible with ARB. Appl. Environ. Microbiol..

[61] Dhariwal A (2017). MicrobiomeAnalyst: A web-based tool for comprehensive statistical, visual and meta-analysis of microbiome data. Nucleic Acids Res..

[62] Jaccard P (1912). The distribution of the flora in the alpine zone. 1. New Phytol..

[63] Bray JR, Curtis JT (1957). An ordination of the upland forest communities of southern Wisconsin. Ecol. Monogr..

[64] Zheng X (2013). A targeted metabolomic protocol for short-chain fatty acids and branched-chain amino acids. Metabolomics.

[65] Morgan XC (2012). Dysfunction of the intestinal microbiome in inflammatory bowel disease and treatment. Genome Biol..

[66] Pinheiro, J. C., *et al.**Nlme: Linear and nonlinear mixed effects models*. R package version 3.1–137 (2018).

