# Supplementary material for: Desacetyl-α-MSH and α-MSH have sex specific interactions with diet to influence mouse gut morphology, metabolites and microbiota
Source: Sci Rep. 2020 Nov 3;10:18957. doi: 10.1038/s41598-020-75786-z (PMC7641164; doi:10.1038/s41598-020-75786-z)
Supplement: Supplementary file 1 — Supplementary Information [file 41598_2020_75786_MOESM1_ESM.pdf]

Desacetyl-α-MSH and α-MSH have sex specific interactions with diet to influence mouse gut morphology, metabolites and microbiota

Bo Sun[1], Tommi Vatanen[2], Thilini N. Jayasinghe[2], Elizabeth McKenzie[2], Rinki Murphy[3,4]*, Justin M. O'Sullivan[2,4]*

1. Department of Physiology, Faculty of Medical and Health Sciences, University of Auckland, Auckland, New Zealand

2. The Liggins Institute, University of Auckland, Auckland, New Zealand

3. School of Medicine, Faculty of Medical and Health Sciences, University of Auckland, Auckland, New Zealand

4. Maurice Wilkins Centre for Molecular Biodiscovery, The University of Auckland, Auckland, New Zealand

*** Co-corresponding authors.**

J. M. O'Sullivan, The Liggins Institute, University of Auckland, 85 Park Road, Grafton 1142, Auckland, New Zealand. Email: justin.osullivan@auckland.ac.nz Ph: 64 9 923 9868

R. Murphy, Department of Medicine, University of Auckland, 85 Park Road, Grafton 1142, Auckland, New Zealand. Email: r.murphy@auckland.ac.nz Ph: 64 9 923 6313

Supplementary data

Table S1. Gut morphology changes in response to a HF diet for male WT and *Pomc*$^{tm1/tm1}$ mice.

Groups	WT LF (N = 8)	WT HF (N = 8)	*Pomc*$^{tm1/tm1}$ LF (N=8)	*Pomc*$^{tm1/tm1}$ HF (N=8)
Small intestine (cm)	35.35 ± 2.91	34.73 ± 1.45	37.03 ± 1.65	37.68 ± 1.27[a]
Colon (cm)	6.834 ± 1.27	7.65 ± 0.59	7.04 ± 1.10	7.58 ± 0.67
Duodenum villus length (µm)	385.54 ± 52.14	391.51 ± 59.78	400.27 ± 62.22	418.70 ± 42.60
Jejunum villus length (µm)	236.03 ± 83.22	284.75 ± 106.61	295.63 ± 64.58	255.00 ± 93.90
Ileum villus length (µm)	194.14 ± 31.29 (N = 7)	203.72 ± 37.18 (N = 7)	210.48 ± 30.50	215.477 ± 46.51 (N = 6)
Duodenum crypt depth (µm)	57.10 ± 4.41	58.42 ± 5.59	62.33 ± 6.23	58.45 ± 4.91
Jejunum crypt depth (µm)	57.00 ± 7.60	58.05 ± 7.96	57.72 ± 4.73	73.33 ± 28.54
Ileum crypt depth (µm)	62.45 ± 9.18 (N = 7)	66.25 ± 8.66 (N = 7)	72.00 ± 5.97	66.04 ± 8.73 (N = 6)
Duodenum V/C	6.76 ± 0.82	6.72 ± 0.97	6.48 ± 1.13	7.18 ± 0.58
Jejunum V/C	4.05 ± 1.00	4.84 ± 1.50	5.14 ± 1.07	3.82 ± 1.62
Ileum V/C	3.15 ± 0.57 (N = 7)	3.09 ± 0.54 (N = 7)	2.92 ± 0.33	3.27 ± 0.58 (N = 6)
Duodenum goblet cells (per mm^2 villus area)	391.65 ± 128.53	433.84 ± 68.11	520.99 ± 155.77	414.05 ± 125.22
Jejunum goblet cells (per mm^2 villus area)	770.89 ± 193.29	672.70 ± 133.45	666.06 ± 157.71	713.94 ± 100.32
Ileum goblet cells (per mm^2 villus area)	1315.37 ± 499.21 (N = 7)	1100.02 ± 270.95	1257.77 ± 285.50	1145.02 ± 178.21 (N = 7)

Gut morphology was measured in male WT and *Pomc*$^{tm1/tm1}$ mice fed on a LF or HF diet for 23 weeks. Data are shown as mean ± SD. Significant differences were determined using two-way ANOVA with Tukey's post-hoc test. a: WT HF v *Pomc*$^{tm1/tm1}$ HF: p = 0.0239 *; LF: Low Fat; HF: High Fat.

Table S2. Gut morphology changes in response to a HF diet for female WT and *Pomc*$^{tm1/tm1}$ mice.

Groups	WT LF (N = 8)	WT HF (N = 7)	*Pomc*$^{tm1/tm1}$ LF (N = 8)	*Pomc*$^{tm1/tm1}$ HF (N = 8)
Small intestine (cm)	33.03 ± 1.76	32.20 ± 0.98	33.61 ± 0.48	33.79 ± 0.79[a]
Colon (cm)	6.73 ± 1.01	6.73 ± 0.52	7.43 ± 0.65	7.14 ± 0.49
Duodenum villus length (µm)	360.68 ± 85.36	333.82 ± 65.04	365.33 ± 70.38	340.46 ± 45.51
Jejunum villus length (µm)	337.89 ± 52.01	263.34 ± 58.74[d]	311.72 ± 78.00	279.82 ± 71.53
Ileum villus length (µm)	190.51 ± 38.55 (N = 6)	173.70 ± 41.83	166.73 ± 54.94	178.16 ± 49.43 (N = 7)
Duodenum crypt depth (µm)	54.80 ± 6.97	53.88 ± 9.46	58.11 ± 6.32	55.21 ± 2.78
Jejunum crypt depth (µm)	53.81 ± 5.18	62.95 ± 7.82[b]	56.51 ± 7.35	56.97 ± 5.04
Ileum crypt depth (µm)	69.15 ± 4.37 (N = 6)	64.35 ± 4.57	65.62 ± 6.00	65.31 ± 6.55
Duodenum V/C	6.55 ± 1.188	6.24 ± 0.87	6.29 ± 1.01	6.18 ± 0.86
Jejunum V/C	6.55 ± 1.19	4.20 ± 0.86[c]	5.47 ± 1.01	4.89 ± 1.20
Ileum V/C	2.75 ± 0.49 (N = 6)	2.68 ± 0.56	2.54 ± 0.79	2.72 ± 0.69
Duodenum goblet cells (per mm^2 villus area)	508.20 ± 115.46	580.46 ± 63.58	441.11 ± 96.94	476.55 ± 61.44
Jejunum goblet cells (per mm^2 villus area)	639.81 ± 121.72	792.00 ± 170.77	741.85 ± 113.50	742.52 ± 96.32
Ileum goblet cells (per mm^2 villus area)	1081.80 ± 178.66 (N = 6)	1268.23 ± 444.95	1105.24 ± 112.05	1117.57 ± 104.26

Gut morphology was measured in female WT and *Pomc*$^{tm1/tm1}$ mice fed on a LF or HF diet for 23 weeks. Data are shown as mean ± SD. Significant differences were determined using either two-way ANOVA with Tukey's post-hoc test, a: WT HF v *Pomc*$^{tm1/tm1}$ HF: $p = 0.0483$ *; b: WT LF v WT HF: $p = 0.0483$ *; c: WT LF v WT HF: $p = 0.0035$ **, or Student's t-test: d: WT LF v WT HF: Student's t-test, $p = 0.022$ **; LF: Low Fat; HF: High Fat.

Table S3. Rarefied sequence depth to same reads in eight pairwise comparisons when investigating the associations between genotype and diet with gut microbiota within sex.

Gender	Pairwise Comparison	Variant	Rarefied sequence depth
Male	WT LF versus WT HF	Diet	24210
Male	*Pomc*$^{tm1/tm1}$ LF versus *Pomc*$^{tm1/tm1}$ HF	Diet	9906
Male	WT LF versus *Pomc*$^{tm1/tm1}$ LF	Genotype	23625
Male	WT HF versus *Pomc*$^{tm1/tm1}$ HF	Genotype	9914
Female	WT LF versus WT HF	Diet	20554
Female	*Pomc*$^{tm1/tm1}$ LF versus *Pomc*$^{tm1/tm1}$ HF	Diet	21831
Female	WT LF versus *Pomc*$^{tm1/tm1}$ LF	Genotype	21867
Female	WT HF versus *Pomc*$^{tm1/tm1}$ HF	Genotype	20466

Table S4. Details on animal grouping, breeding pair and quality-filtered sequence reads.

Animal_ID	Gender[σ]	Genotype[#]	Diet[ǂ]	CageID[δ]	BreedingPairID[@]	MotherID	Quality-filtered Sequence Reads
WT 8AM1	M	WT	LF	C1	BP3	WT89BF4	48159
WT 16AM1	M	WT	LF	C1	BP8	WT93BF2	45769
WT 4AM1	M	WT	LF	C1	BP1	WT79EF3	40475
WT 14AM4	M	WT	LF	C1	BP6	WT94AF7	61808
WT 8AM2	M	WT	LF	C2	BP3	WT89BF4	51590
WT 16AM2	M	WT	LF	C2	BP8	WT93BF2	36941
WT 4AM2	M	WT	LF	C2	BP1	WT79EF3	44296
WT 14AM5	M	WT	LF	C2	BP6	WT94AF7	59415
WT 8AM4	M	WT	HF	C4	BP3	WT89BF4	24582
WT 17AM1	M	WT	HF	C4	BP9	WT96BF3	39328
WT 14AM1	M	WT	HF	C4	BP6	WT94AF7	26841
WT18AM2	M	WT	HF	C4	BP10	WT94BF1	26116
WT 15AM1	M	WT	HF	C5	BP7	WT87EF1	26315
WT 17AM2	M	WT	HF	C5	BP9	WT96BF3	24281
WT 14AM2	M	WT	HF	C5	BP6	WT94AF7	33126
WT18AM3	M	WT	HF	C5	BP10	WT94BF1	26515
Hom 38AM1	M	*Pomc*$^{tm1/tm1}$	LF	C7	BP13	Hom86DF1	50600
Hom 45AM1	M	*Pomc*$^{tm1/tm1}$	LF	C7	BP18	Hom90AF1	24278
Hom 41AM2	M	*Pomc*$^{tm1/tm1}$	LF	C7	BP14	Hom94AF3	24098
Hom 43AM3	M	*Pomc*$^{tm1/tm1}$	LF	C7	BP16	Hom86EF2	47121
Hom 44AM1	M	*Pomc*$^{tm1/tm1}$	LF	C8	BP17	Hom91BF2	31309
Hom 46AM1	M	*Pomc*$^{tm1/tm1}$	LF	C8	BP19	Hom96BF2	50604
Hom 41AM3	M	*Pomc*$^{tm1/tm1}$	LF	C8	BP14	Hom94AF3	40729
Hom43AM4	M	*Pomc*$^{tm1/tm1}$	LF	C8	BP16	Hom86EF2	44830
Hom 44AM3	M	*Pomc*$^{tm1/tm1}$	HF	C10	BP17	Hom91BF2	24960
Hom 36AM1	M	*Pomc*$^{tm1/tm1}$	HF	C10	BP11	Hom79EF1	36157
Hom 41AM5	M	*Pomc*$^{tm1/tm1}$	HF	C10	BP14	Hom94AF3	43845
Hom 37AM1	M	*Pomc*$^{tm1/tm1}$	HF	C10	BP12	Hom88BF4	33168
Hom 44AM4	M	*Pomc*$^{tm1/tm1}$	HF	C11	BP17	Hom91BF2	39083
Hom 36AM2	M	*Pomc*$^{tm1/tm1}$	HF	C11	BP11	Hom79EF1	9899
Hom 43AM1	M	*Pomc*$^{tm1/tm1}$	HF	C11	BP16	Hom86EF2	18217
Hom 42AM1	M	*Pomc*$^{tm1/tm1}$	HF	C11	BP15	Hom94AF1	57575
WT 8AF1	F	WT	LF	C13	BP3	WT89BF4	35088
WT 16AF4	F	WT	LF	C13	BP8	WT93BF2	45355
WT 14AF3	F	WT	LF	C13	BP6	WT94AF7	48250
WT 7AF2	F	WT	LF	C13	BP2	WT88BF2	27671
WT 15AF1	F	WT	LF	C14	BP7	WT87EF1	28983

WT 17AF1	F	WT	LF	C14	BP9	WT96BF3	36898
WT 18AF1	F	WT	LF	C14	BP10	WT94BF1	54461
WT 7AF3	F	WT	LF	C14	BP2	WT88BF2	22136
WT 16AF1	F	WT	HF	C16	BP8	WT93BF2	20521
WT 4AF2	F	WT	HF	C16	BP1	WT79EF3	38994
WT 18AF3	F	WT	HF	C16	BP10	WT94BF1	45740
WT 9AF2	F	WT	HF	C16	BP4	WT85CF2	34483
WT 16AF2	F	WT	HF	C17	BP8	WT93BF2	24706
WT 18AF4	F	WT	HF	C17	BP10	WT94BF1	42899
WT 10AF1	F	WT	HF	C17	BP5	WT85CF1	43381
Hom 38AF1	F	$Pomc^{tm1/tm1}$	LF	C19	BP13	Hom86DF1	21822
Hom 44AF2	F	$Pomc^{tm1/tm1}$	LF	C19	BP17	Hom91BF2	58365
Hom 46AF2	F	$Pomc^{tm1/tm1}$	LF	C19	BP19	Hom96BF2	43483
Hom 36AF4	F	$Pomc^{tm1/tm1}$	LF	C19	BP11	Hom79EF1	42869
Hom 38AF2	F	$Pomc^{tm1/tm1}$	LF	C20	BP13	Hom86DF1	44503
Hom 44AF3	F	$Pomc^{tm1/tm1}$	LF	C20	BP17	Hom91BF2	33578
Hom 46AF3	F	$Pomc^{tm1/tm1}$	LF	C20	BP19	Hom96BF2	47845
Hom 36AF5	F	$Pomc^{tm1/tm1}$	LF	C20	BP11	Hom79EF1	29316
Hom 38AF4	F	$Pomc^{tm1/tm1}$	HF	C22	BP13	Hom86DF1	59100
Hom 45AF1	F	$Pomc^{tm1/tm1}$	HF	C22	BP18	Hom90AF1	48964
Hom 36AF1	F	$Pomc^{tm1/tm1}$	HF	C22	BP11	Hom79EF1	33802
Hom 41AF1	F	$Pomc^{tm1/tm1}$	HF	C22	BP14	Hom94AF3	29386
Hom 38AF5	F	$Pomc^{tm1/tm1}$	HF	C23	BP13	Hom86DF1	30126
Hom 45AF2	F	$Pomc^{tm1/tm1}$	HF	C23	BP18	Hom90AF1	33660
Hom 36AF2	F	$Pomc^{tm1/tm1}$	HF	C23	BP11	Hom79EF1	36555

σ: M: Male; F: Female; #: WT: Wild Type; ≁: LF: Low Fat; HF: High Fat; δ: C: Cage; @: BP: Breeding Pair.

Table S5. Dried fecal weight samples and SCFAs and BCAAs concentrations analyzed by MassOmics (before normalization by fecal weights) for male WT and *Pomc*$^{tm1/tm1}$ mice.

Sample ID	Feces wet weight (mg)	Feces dried weight (mg)	Acetic acid µg/ml	Butyric acid µg/ml	Propionic acid µg/ml	L-leucine µg/ml	L-isoleucine µg/ml	L-valine µg/ml
WT 8AM1	57	30	7.9	2.2	2.3	13.1	9.1	10.8
WT 16AM1	85	40	102.5	46.3	35.0	33.2	22.0	28.6
WT 4AM1	84	38	29.7	14.4	15.7	29.7	20.2	23.1
WT 14AM4	83	38	29.9	31.3	20.5	17.5	14.2	16.7
WT 8AM2	98	40	35.4	52.2	23.7	23.4	18.4	19.1
WT 16AM2	52	23	16.8	11.0	9.8	1.8	2.4	2.5
WT 4AM2	89	43	13.5	26.2	9.9	8.8	11.9	9.4
WT 14AM5	101	51	43.1	25.1	22.6	8.9	7.7	7.8
WT 8AM4	69	31	31.4	17.8	11.3	12.7	9.4	12.9
WT 17AM1	81	48	46.1	7.2	8.4	19.8	14.5	17.7
WT 14AM1	82	44	79.8	32.5	15.1	45.0	34.0	36.8
WT18AM2	59	33	30.1	7.9	9.5	16.9	11.7	12.7
WT 15AM1	24	12	8.0	26.8	0.43	2.1	3.8	3.4
WT 17AM2	51	24	8.5	15.4	3.1	6.0	5.5	5.5
WT 14AM2	75	37	11.4	71.8	13.9	14.6	14.1	13.2
WT18AM3	37	19	10.1	5.6	2.9	4.4	4.2	5.0
Hom 38AM1	68	33	10.6	33.0	8.1	6.3	6.0	5.3
Hom 45AM1	114	61	32.6	17.5	13.8	27.2	20.9	22.7
Hom 41AM2	77	31	32.1	51.2	24.0	9.9	10.7	12.3
Hom 43AM3	90	40	24.5	27.2	18.9	10.7	9.3	9.8
Hom 44AM1	100	41	49.6	58.0	26.4	30.7	25.1	29.2
Hom 46AM1	64	23	32.1	60.7	24.6	0.1	2.0	2.0
Hom 41AM3	67	26	28.3	71.9	27.5	3.3	4.4	3.8
Hom43AM4	90	41	23.4	47.7	17.9	6.1	8.2	6.9
Hom 44AM3	77	33	54.4	50.5	25.5	15.0	14.6	13.5
Hom 36AM1	85	39	35.4	39.4	13.1	19.0	15.7	17.0
Hom 41AM5	98	37	106.1	68.7	33.2	54.4	33.4	39.6
Hom 37AM1	80	21	23.0	20.9	8.7	7.3	7.2	8.7
Hom 44AM4	51	25	10.3	12.9	4.1	7.1	5.7	6.2
Hom 36AM2	83	34	31.1	45.2	26.9	4.9	5.4	5.8
Hom 43AM1	52	27	22.0	20.0	11.6	22.2	16.7	20.5
Hom 42AM1	63	29	27.7	34.7	10.3	6.7	6.0	8.0

Table S6. Dried fecal weight samples and SCFAs and BCAAs concentrations analyzed by MassOmics (before normalization by fecal weights) for female WT and *Pomc*$^{tm1/tm1}$ mice.

Sample ID	Feces wet weight (mg)	Feces dried weight (mg)	Acetic acid µg/ml	Butyric acid µg/ml	Propionic acid µg/ml	L-leucine µg/ml	L-isoleucine µg/ml	L-valine µg/ml
WT8AF1	42	15	9.8	1.0	undetectable	4.9	4.3	4.9
WT16AF4	21	7	17.0	5.6	5.6	0.3	0.3	1.0
WT14AF3	36	16	5.3	2.8	3.8	1.6	1.2	1.4
WT7AF2	43	20	8.3	5.0	undetectable	1.3	1.1	1.8
WT15AF1	38	18	6.3	3.7	4.3	2.0	1.6	2.0
WT17AF1	52	24	16.7	11.9	10.4	2.1	1.7	2.2
WT18AF1	7	6	2.1	5.2	0.8	undetectable	0.2	0.1
WT7AF3	38	21	30.4	10.5	undetectable	3.7	3.3	4.1
WT16AF1	25	14	11.7	2.3	6.9	1.5	1.2	1.6
WT4AF2	27	10	6.4	3.5	1.4	2.0	1.8	2.1
WT18AF3	115	43	92.7	26.4	23.5	26.1	15.7	18.1
WT9AF2	43	21	13.0	5.2	7.5	1.5	1.5	2.2
WT16AF2	24	11	6.5	2.7	1.3	0.2	0.1	0.6
WT18AF4	28	15	9.4	2.7	3.9	undetectable	0.0	0.4
WT10AF1	28	17	9.9	3.8	8.4	3.2	2.2	3.3
Hom38AF	30	18	5.8	10.9	4.0	4.8	5.7	5.3
Hom44AF	35	15	10.2	7.2	7.7	4.6	3.6	4.5
Hom46AF	61	28	14.2	19.3	8.2	7.7	7.3	6.9
Hom36AF	46	23	11.3	18.4	7.2	17.0	13.8	15.3
Hom38AF	32	16	5.9	4.9	4.2	2.2	2.4	3.4
Hom44AF	40	22	7.8	5.2	0.7	1.9	1.5	1.7
Hom46AF	11	6	1.8	1.6	undetectable	undetectable	undetectable	undetectable
Hom36AF	29	15	6.7	2.3	1.1	undetectable	0.1	0.5
Hom38AF	44	23	14.9	3.7	undetectable	10.4	6.7	8.8
Hom36AF	32	15	8.3	3.9	7.1	5.2	4.0	4.7
Hom41AF	27	15	5.5	2.1	undetectable	2.1	1.9	2.3
Hom38AF	16	7	5.4	1.2	1.9	0.2	0.4	1.2
Hom45AF	14	10	2.0	0.7	undetectable	1.9	1.2	1.6
Hom36AF	44	23	7.4	3.1	3.5	6.5	4.9	6.4
Hom41AF	17	8	5.9	1.5	1.3	2.5	2.1	3.3

Supplementary Figure Legends

Figure S1. Principal coordinates analysis (PCoA) plots showing the β-diversity clustering patters of male samples. A, WT mice fed on HF and LF diets; **B**, *Pomc*$^{tm1/tm1}$ mice fed on HF and LF diets; **C**, WT mice and *Pomc*$^{tm1/tm1}$ mice fed on LF diet; **D**, WT mice and *Pomc*$^{tm1/tm1}$ mice fed on HF diet. PCoA plots were based on Bray-Curtis dissimilarities. Cage ID was labelled. WT: wild type; LF: low fat; HF: high fat.

Figure S2. Principal coordinates analysis (PCoA) plots showing the β-diversity clustering patters of female samples. A, WT mice fed on HF and LF diets; **B**, *Pomc*$^{tm1/tm1}$ mice fed on HF and LF diets; **C**, WT mice and *Pomc*$^{tm1/tm1}$ mice fed on LF diet; **D**, WT mice and *Pomc*$^{tm1/tm1}$ mice fed on HF diet. PCoA plots were based on Bray-Curtis dissimilarities. Cage ID was labelled. WT: wild type; LF: low fat; HF: high fat.

Figure S3. Relative abundance of main phyla and genera. Bar charts showing the relative abundance of the main phyla within the cecal microbiota in male (**A**) and female (**B**), and the relative abundance of the genera within the cecal microbiota in male (**C**) and female (**D**). *Pomc*$^{tm1/tm1}$ and WT mice (n = 7 or 8 mice per group, two cages per group). Each taxon representing > 1% of the relative abundance per sample is indicated by a different color. WT: wild type; LF: low fat; HF: high fat.

Figure S4. Fecal SCFAs concentrations in males. SCFAs were measured using PCF/GC-MS method, and the final concentrations were normalized by dried feces weights; **A,** acetic acids; **B**, propionic acid; **C**, butyric acid; Data are mean ± S.E.M. The differences were determined by using linear mixed-effects models, *, $p < 0.05$.

Figure S5. Fecal BCAAs concentrations. BCAAs were measured using PCF/GC-MS method, and the final concentrations were normalized by dried feces weights; **A, C, E**: male L-leucin, L-isoleucine and L-valine; **B, D, F**: female L-leucin, L-isoleucine and L-valine; Data are mean ± S.E.M. The differences were determined by using linear mixed-effects models.

Figure S6. Rarefaction curve based on phylogenetic diversity (PD) showed sequence depth almost reached saturation for each sample. A flat line would indicate that the analysis of more sequences would not be able to detect more species (OTUs at 97% similarity), which reached saturation. Each sample is indicated by different color.

Figure S1.

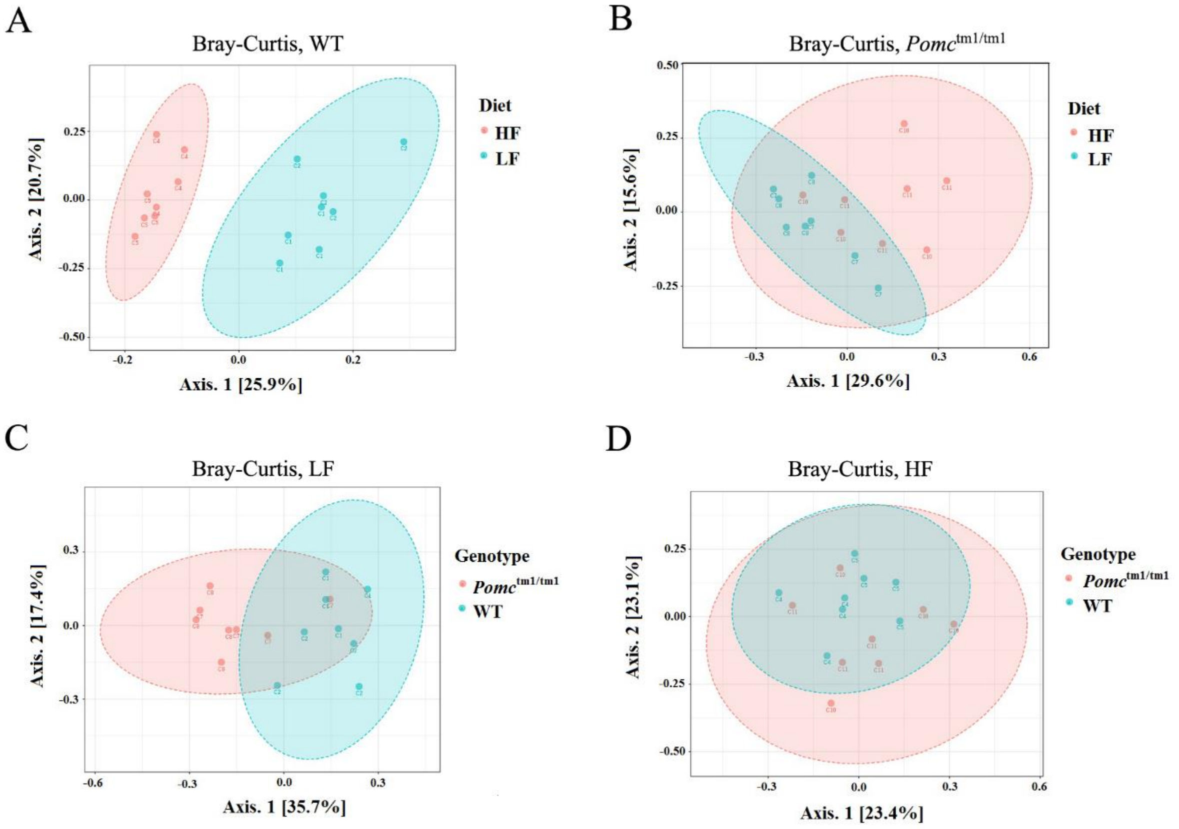
A
Bray-Curtis, WT
Axis. 2 [20.7%]
Axis. 1 [25.9%]
Diet
HF
LF
B
Bray-Curtis, Pomctm1/tm1
Axis. 2 [15.6%]
Axis. 1 [29.6%]
Diet
HF
LF
C
Bray-Curtis, LF
Axis. 2 [17.4%]
Axis. 1 [35.7%]
Genotype
Pomctm1/tm1
WT
D
Bray-Curtis, HF
Axis. 2 [23.1%]
Axis. 1 [23.4%]
Genotype
Pomctm1/tm1
WT

Figure S2.

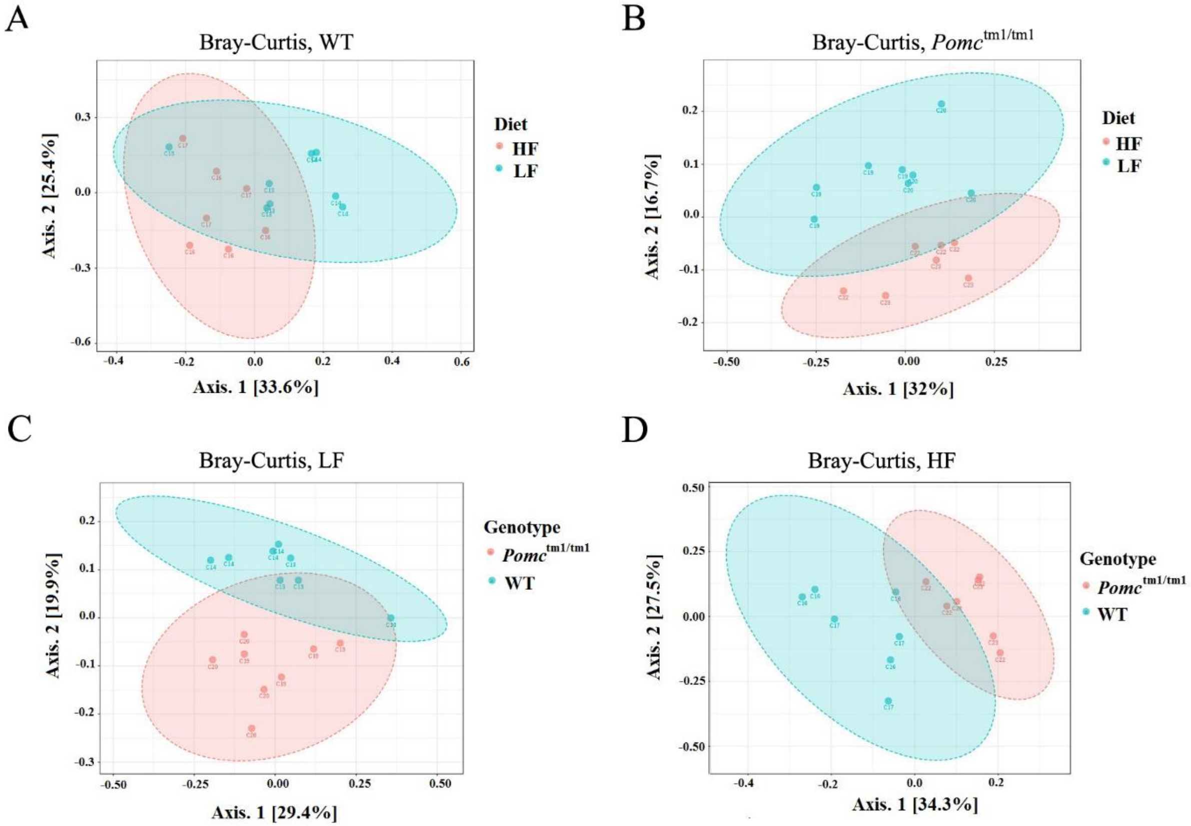
A
Bray-Curtis, WT
Axis. 2 [25.4%]
Axis. 1 [33.6%]
Diet
HF
LF
B
Bray-Curtis, Pomc tm1/tm1
Axis. 2 [16.7%]
Axis. 1 [32%]
Diet
HF
LF
C
Bray-Curtis, LF
Axis. 2 [19.9%]
Axis. 1 [29.4%]
Genotype
Pomc tm1/tm1
WT
D
Bray-Curtis, HF
Axis. 2 [27.5%]
Axis. 1 [34.3%]
Genotype
Pomc tm1/tm1
WT

Figure S3.

A. Male phyla

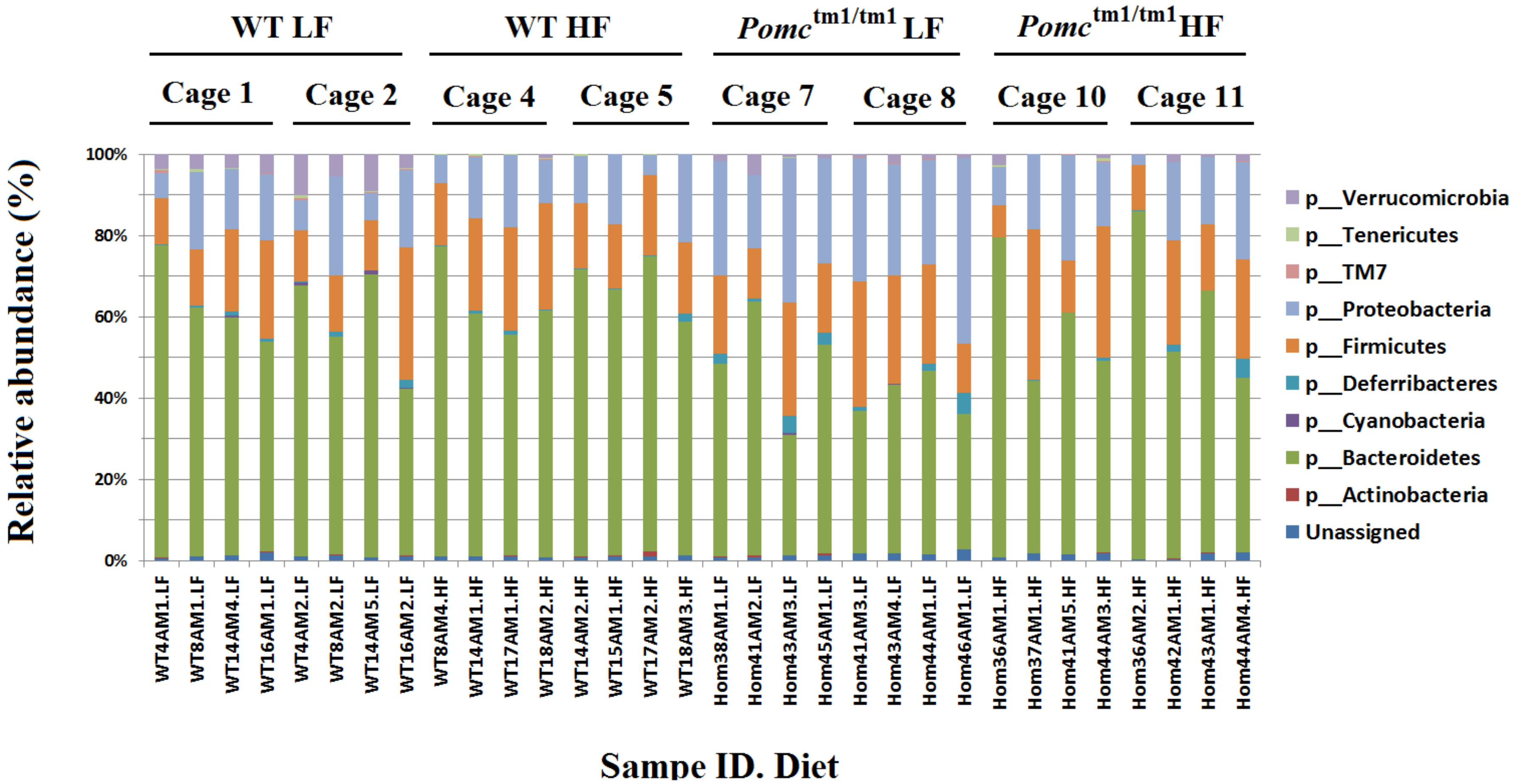

B. Female phyla

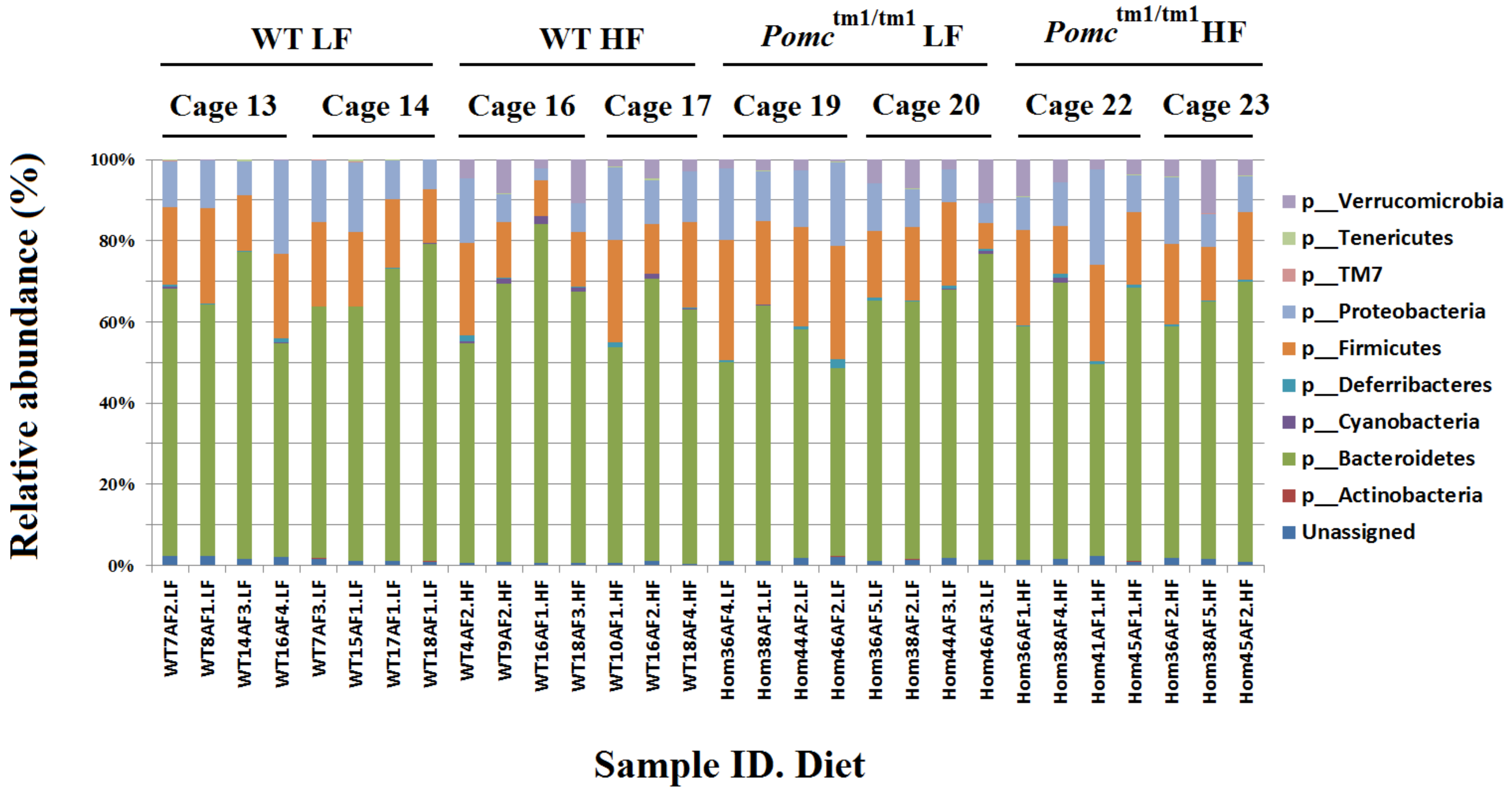

C. Male genera

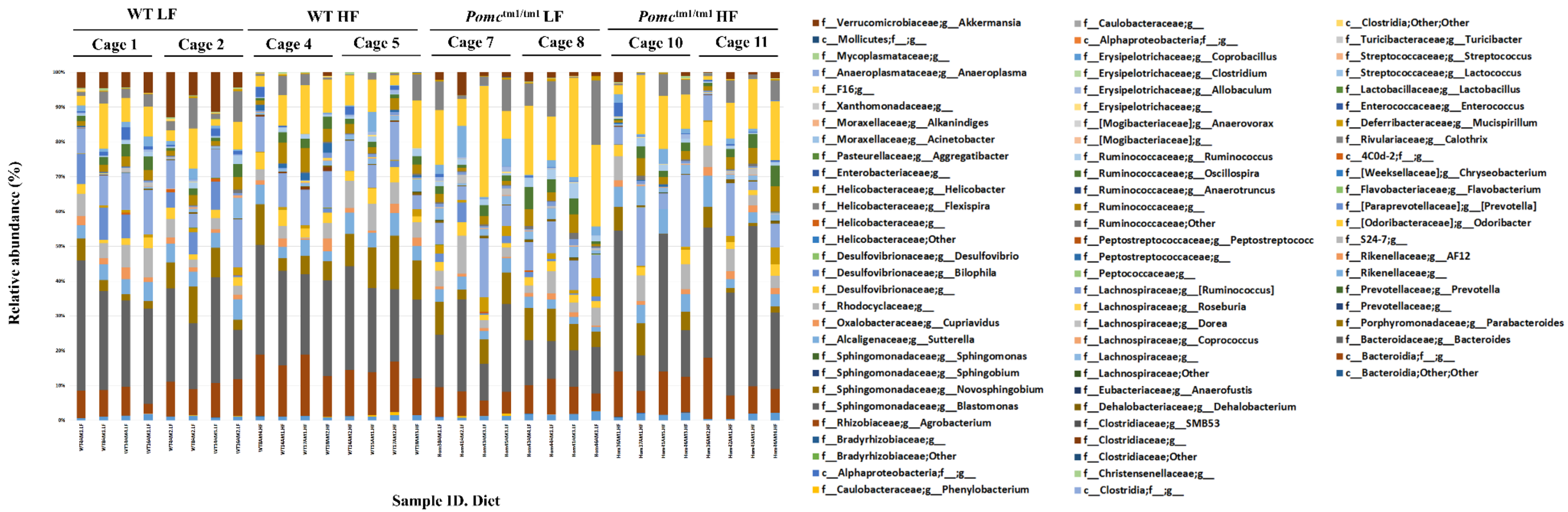

D. Female genera

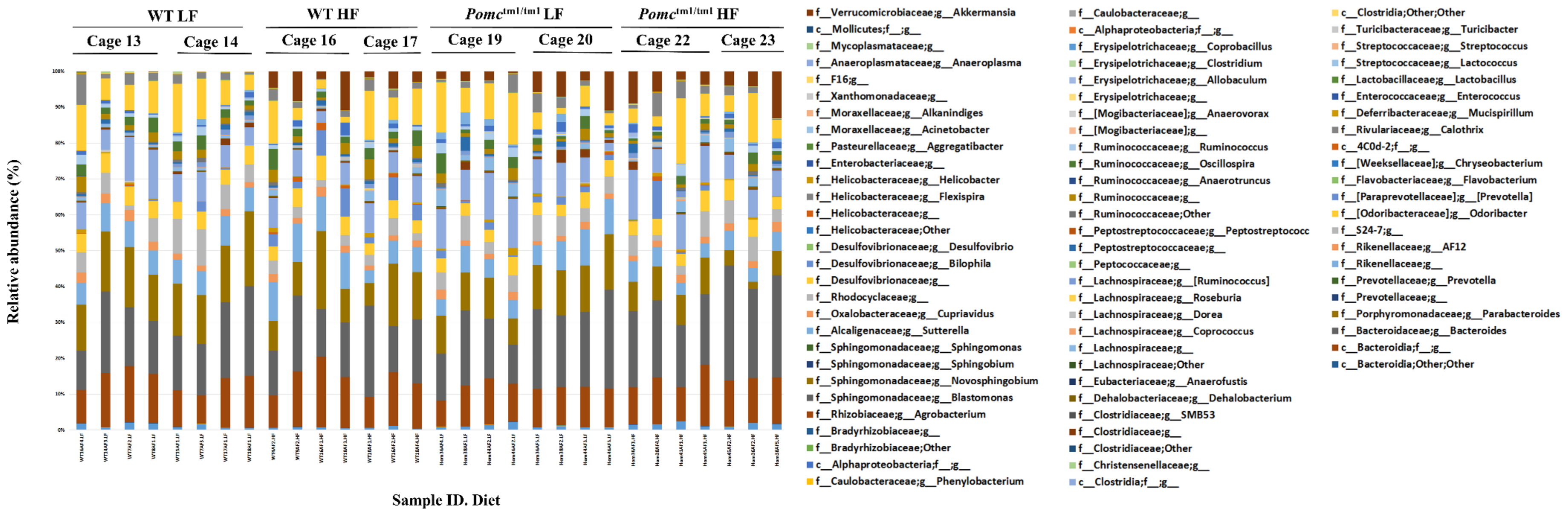

Figure S4.

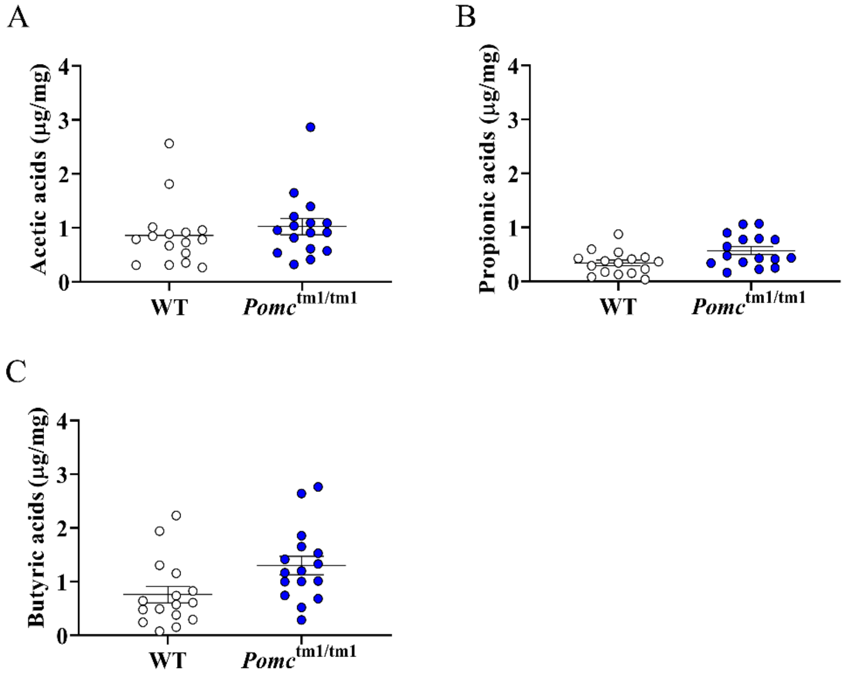

A
Acetic acids (µg/mg)
4
3
2
1
0
WT
Pomc tm1/tm1
B
Propionic acids (µg/mg)
4
3
2
1
0
WT
Pomc tm1/tm1
C
Butyric acids (µg/mg)
4
3
2
1
0
WT
Pomc tm1/tm1

Figure S5.

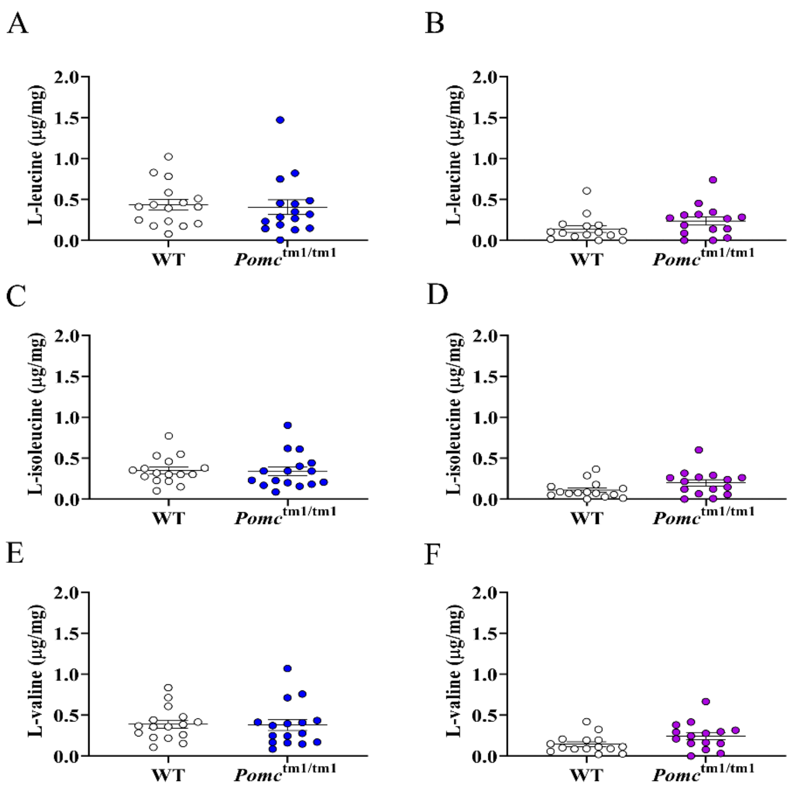
A
B
C
D
E
F
L-leucine (µg/mg)
L-isoleucine (µg/mg)
L-valine (µg/mg)
2.0
1.5
1.0
0.5
0.0
WT
Pomc tm1/tm1

Figure S6.

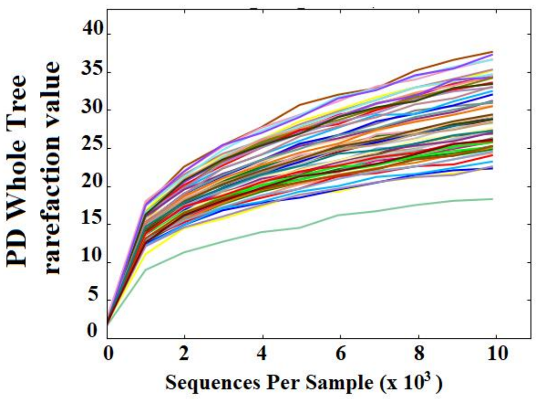
PD Whole Tree
rarefaction value
40
35
30
25
20
15
10
5
0
0
2
4
6
8
10
Sequences Per Sample (x 10³)
